# Effectiveness and Economic Evaluation of Polyene Phosphatidyl Choline in Patients With Liver Diseases Based on Real-World Research

**DOI:** 10.3389/fphar.2022.806787

**Published:** 2022-03-07

**Authors:** Jian-Gao Fan, Ying Li, Ze Yu, Xing-Xian Luo, Ping Zheng, Xin Hao, Ze-Yuan Wang, Fei Gao, Guo-Qing Zhang, Wan-Yu Feng

**Affiliations:** ^1^ Department of Gastroenterology, Xin Hua Hospital Affiliated to Shanghai Jiao Tong University School of Medicine, Shanghai, China; ^2^ Department of Pharmacy, Shanghai Pulmonary Hospital, Tongji University, Shanghai, China; ^3^ Beijing Medicinovo Technology Co. Ltd., Beijing, China; ^4^ School of Life Science and Biopharmaceutics, Shenyang Pharmaceutical University, Shenyang, China; ^5^ Department of Pharmacy, Nanfang Hospital, Southern Medical University, Guangzhou, China; ^6^ Dalian Medicinovo Technology Co. Ltd., Dalian, China; ^7^ Department of Pharmacy, Eastern Hepatobiliary Surgery Hospital, Second Military Medical University, Shanghai, China; ^8^ Department of Pharmacy, Peking University People’s Hospital, Beijing, China

**Keywords:** polyene phosphatidyl choline, liver protective drug, liver disease, real-world research, effectiveness, economic evaluation

## Abstract

**Aims:** Liver disease has high prevalence, number, and disease burden in China, and polyene phosphatidyl choline (PPC) is a widely used liver protective drug. We aim to explore the effectiveness and economy of PPC in patients with liver diseases based on real-world research and compare with other hepatoprotective drugs.

**Methods:** This is a “three-phase” study from three medical centers, including descriptive study of patients using PPC injection, self-control case study of patients using PPC injection, and specific-disease cohort study of patients using PPC injection or control drugs. The major measurements of liver function for effectiveness analysis were the alanine transaminase (ALT) level changes and recovery rate. The main statistical methods were Wilcoxon signed rank test, *χ* ^2^ test, and Mann–Whitney *U* test. Propensity score matching was applied to reduce bias. Cost-effectiveness analysis, cost minimization analysis, and sensitivity analysis were used for economic evaluation.

**Results:** PPC alone or in combination with glutathione and magnesium isoglycyrrhizinate shows less total hospitalization cost (*p* < 0.05) and smaller cost-effectiveness ratio and was effective in protecting liver function, especially in patients with liver transplantation or postoperation of nontumor liver disease (ALT decreased significantly after PPC treatment; *p* < 0.05). Glutathione and magnesium isoglycyrrhizinate combined with PPC could enhance the protective function of liver.

**Conclusion:** PPC was an effective and economic liver protective drug in patients with specific liver diseases, and PPC could enhance the liver protective function of glutathione and magnesium isoglycyrrhizinate.

## Introduction

Compared with developed countries such as Western Europe and North America, the prevalence rate and disease burden of liver disease in China are huge, and the trend is still increasing yearly. The application of liver protection drugs is important to reduce the incidence of liver injury and repair liver tissue. Clinical commonly used liver protective drugs include polyene phosphatidyl choline (PPC), glutathione, and magnesium isoglycyrrhizinate in China. PPC is a major component of phospholipids, extracted from soy, and rich in polyunsaturated fatty acids, such as linoleic, linolenic, and oleic acids (Xiang et al., 2013; Feng et al., 2020). Compared with other liver protective drugs, PPC is indicated for a broad range of conditions, including viral hepatitis, drug-induced liver injury, and nonalcoholic steatohepatitis (Lieber et al., 1997; Cao et al., 2016; Committee of the treatmen, 2017). One important component in PPC is phosphatidylcholine, which forms the organelle membranes and the cell membrane. (Feng et al., 2020) Previous animal studies have proven that PPC can repair damaged membranes of hepatocytes and relieve hepatic necroinflammation (Okiyama et al., 2009; Cao et al., 2016). In the clinical, PPC is commonly used alone or in combination with other liver protective drugs, such as glutathione, magnesium isoglycyrrhizinate, ademetionine, and acetylcysteine, to protect liver function (Mu et al., 2018; Wang and Chen, 2020). Understanding the current situation, effectiveness and economic evaluation of PPC could help promote the application of PPC in patients with liver disease.

With the rapid development of information technology, real-world evidence from medical records has become an important data source for clinical research. Real-world research is rooted in clinical practice and comes from a wide range of sources, including hospitalization records, laboratory examination, images, and follow-up records during diagnosis and treatment. There were multiple studies based on real-world evidence demonstrating effectiveness and economy of different drugs, such as erenumab for headache, infliximab for Crohn disease, and apatinib for metastatic colorectal cancer (Gou et al., 2018; Kanaan et al., 2020; Shi et al., 2020). Our aims were to explore the effectiveness and economy of PPC in patients with liver diseases based on real-world research, compare it with other hepatoprotective drugs (alone or combination medication), and evaluate the application of PPC in specific-disease cohorts, in order to optimal regimen therapy in clinical to treat hepatopathies.

## Materials and Methods

### Study Design and Population

**FIGURE 1:**
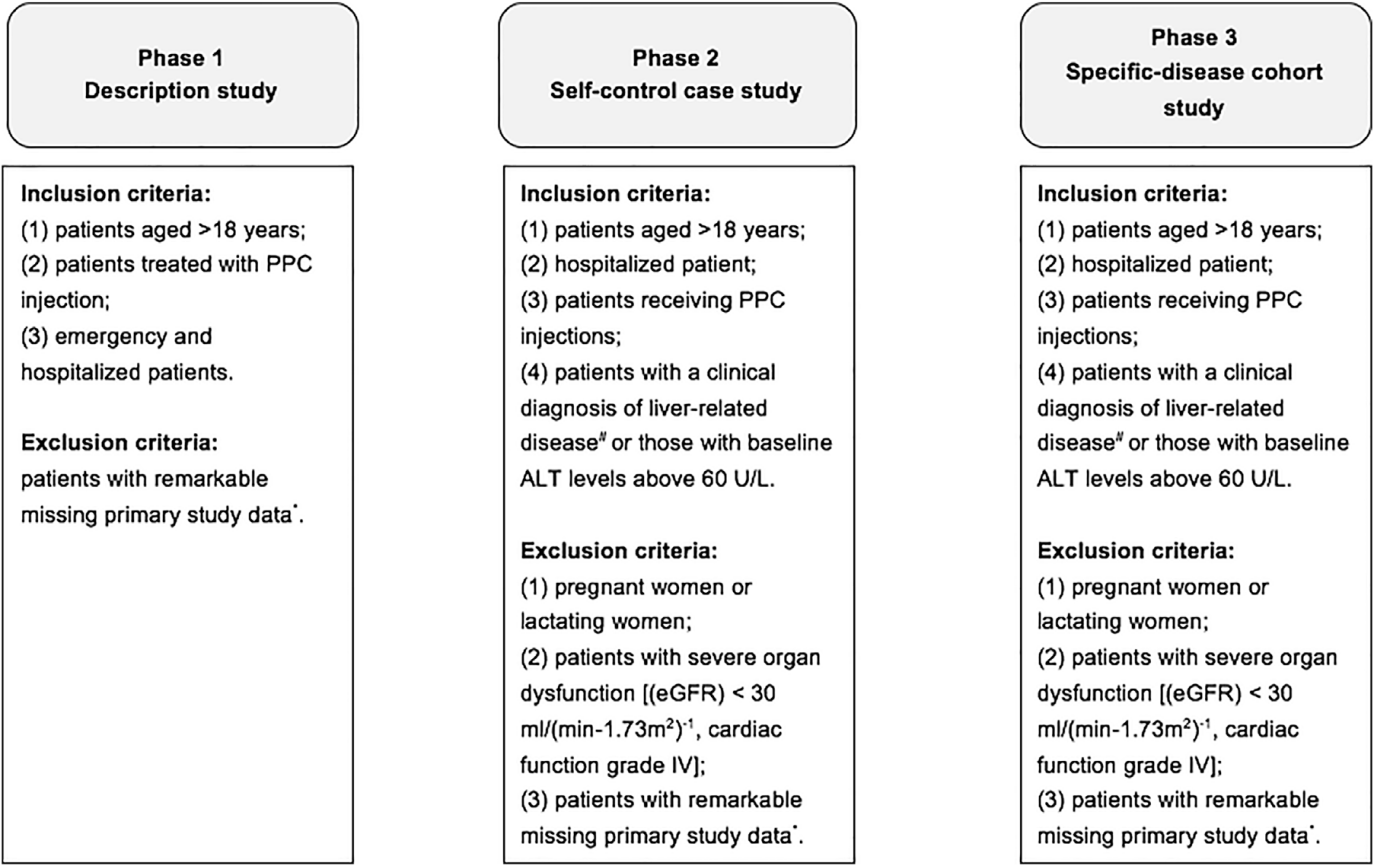
The workflow of the whole study. Notes: * indicates patient ID, age, gender, and information related to disease diagnosis. # indicates various types of hepatitis, cirrhosis, alcoholic liver disease, nonalcoholic fatty liver disease, drug-induced liver disease, autoimmune liver disease, liver fibrosis, hepatic encephalopathy, hepatolenticular degeneration, liver cancer, liver transplantation, hepatobiliary surgery, common liver- occupying lesions, and other causes of abnormal liver function. Abbreviations: eGFR, estimated glomerular filtration rate.

Data were obtained from Xinhua Hospital Affiliated to Shanghai Jiaotong University School of Medicine and Shanghai Eastern Hepatobiliary Surgery Hospital from January 1, 2015, to January 1, 2020, and Nanfang Hospital of Southern Medical University from January 1, 2017, to January 1, 2020. The whole study was a “three-phase” design, and the study workflow is presented in Figure 1. To be specific, phase I was a descriptive study of patients using PPC injection, phase II was a self-control case study of patients using PPC injection, and phase III was a specific-disease cohort study of patients using PPC injection and/or control drugs (other liver protective drugs). The primary measurements of effectiveness were the level changes of serum alanine transaminase (ALT) relative to baseline (ALT change) and the proportion of ALT that had previously exceeded the upper limit of normal range decrease to less than 40 U/L after treatment (ALT recovery), and secondary measurements of effectiveness were the level changes of serum aspartate transferase (AST) relative to baseline (AST change) and the proportion of AST that had previously exceeded the upper limit of normal range decrease to less than 40 U/L after treatment (AST recovery), and the level changes of total bilirubin (TBil) relative to baseline (TBil change) and the proportion of TBil that had previously exceeded the upper limit of normal range decreased to less than 17.1 μmol/L after treatment (TBil recovery). Furthermore, the economic evaluation methods include cost-effectiveness analysis, cost minimization analysis, and sensitivity analysis, mainly used in phase III.

The inclusion criteria in phase I were as follows: (1) patients aged >18 years, (2) patients treated with PPC injection, and (3) emergency and hospitalized patients. Exclusion criteria were as follows: patients with remarkable missing primary study data, including patient ID, age, gender, and information related to disease diagnosis. In phases II and III, the inclusion criteria included: (1) patients aged >18 years, (2) hospitalized patient, (3) patients receiving PPC injections, and (4) patients with a clinical diagnosis of liver-related disease (including various types of hepatitis, cirrhosis, alcoholic liver disease, nonalcoholic fatty liver disease (NAFLD), drug-induced liver disease, autoimmune liver disease, liver fibrosis, hepatic encephalopathy, hepatolenticular degeneration, liver cancer, liver transplantation, hepatobiliary surgery, common liver occupying lesions, and other causes of abnormal liver function test) or those with baseline ALT levels greater than 60 U/L. The exclusion criteria in phases II and III include the following: (1) pregnant women or lactating women; (2) patients with severe organ dysfunction [such as estimated glomerular filtration rate (eGFR) <30 mL/(min-1.73 m^2^)^−1^, cardiac function grade IV]; and (3) patients with remarkable missing primary study data, including patient ID, age, gender, information related to disease diagnosis, and information related to the use of PPC injection.

### Diagnostic Classification Criteria for Liver Diseases

According to the discharge diagnosis in the hospitalization record, the first clinical diagnosis was defined as the most severe diagnosis in the discharge record, and one hospitalization corresponded to one first clinical diagnosis. The discharge diagnosis was ranked in the order of disease severity as (“=” means equal level of severity): (1) postoperation of tumor/liver transplantation; (2) cirrhosis (including partial liver atrophy and schistosomiasis liver disease) = hepatic encephalopathy (including coma); (3) viral hepatitis = drug-induced liver injury = autoimmune liver disease = alcoholic liver disease = NAFLD; (4) abnormal liver function test (abnormal liver function of unknown etiology, hepatitis, liver insufficiency and liver failure) = hepatic vascular diseases = space-occupying lesions/postoperative (mainly benign and unknown diagnosis) = nonneoplastic diseases of the biliary tract (including biliary operations and postoperations) = others (space-occupying lesions, porta hepatis narrow, Wilson disease, and multiple organ function failure of uncertain etiology). When patients had diseases of the same severity, the one ranked first in the above should prevail (the severity ranking of the diseases is a theoretical order, and clinical conditions should also be referred to for specific situations). Moreover, only the most severe stage was recorded when different liver disease stages occurred simultaneously, such as the concurrence of liver cancer and cirrhosis, and the first clinical diagnosis was counted as liver cancer.

### Phase I

Phase I was a descriptive study of patients receiving PPC injections. All hospitalization records and initial hospitalization records treated with PPC injection were used to describe (1) demographic information, such as age and gender; (2) disease-related information, including patient’s first clinical diagnosis, liver disease spectrum of discharge diagnosis in the hospitalization records (extracting all diagnoses related to the word “hepatic” or “liver”), basic chronic diseases (including hypertension, diabetes and hyperlipidemia), surgery situation (including non-surgery; surgery may affect liver function, and no significant/unknown effect of surgery on liver function), and PPC combination drug therapy; and (3) economic information, such as total days of using PPC, hospitalization days, and total treatment cost.

### Phase II

Phase II was a self-control case study of patients using PPC injection. In this part, all hospitalization records were used for analysis. Subgroup analysis was conducted according to whether the liver disease spectrum was postoperation of tumor/liver transplantation, postoperation of non-tumor/liver transplantation, viral hepatitis, liver cirrhosis, and abnormal liver function. Main descriptions were as follows: (1) demographic information, such as age and gender; (2) disease-related information, including patient’s first clinical diagnosis, liver disease spectrum of discharge diagnosis in the hospitalization records (extracting all diagnoses related to the word “hepatic” or “liver”), surgery situation (including non-surgery, surgery may affect liver function, and no significant/unknown effect of surgery on liver function), basic chronic diseases (including hypertension, diabetes and hyperlipidemia); (3) economic information, such as total days of using PPC, hospitalization days, and total treatment cost; and (4) effectiveness analysis, such as ALT/AST/TBil levels and their changes relative to baseline after treatment.

### Phase III

Phase III was a case–control study among disease-specific cohorts (postoperation of tumor/liver transplantation, postoperation of non-tumor/liver transplantation, viral hepatitis, liver cirrhosis, and abnormal liver function); the involved medication groups included PPC injection alone, magnesium isoglycyrrhizinate injection alone, glutathione injection alone, PPC + glutathione injection, PPC + magnesium isoglycyrrhizinate injection, PPC + glutathione + magnesium isoglycyrrhizinate injection, and glutathione + magnesium isoglycyrrhizinate injection. Because of the small sample size or the lack data of control group, the difference between case and control groups of other drug combinations in phase III could not be compared. In this part, all hospitalization records were used for analysis. Main descriptions included (1) demographic information; (2) disease-related information, including patient’s first clinical diagnosis, liver disease spectrum of discharge diagnosis in the hospitalization records (extracting all diagnoses related to the word “hepatic” or “liver”), basic chronic diseases (including hypertension, diabetes and hyperlipidemia), and PPC combination drug therapy; (3) effectiveness analysis, such as ALT/AST/TBil levels and their changes relative to baseline after treatment, as well as the intergroup difference analysis; and (4) economic analysis, such as cost minimization analysis, cost-effectiveness analysis, and sensitivity analysis.

### Statistical Analysis

All statistical tests were conducted on a two-sided basis, and *p* < 0.05 was considered statistically significant (unless otherwise noted). Quantitative data were described by number, mean, standard deviation, median, minimum and maximum, and the upper and lower quartiles. Categorical data were described by the number and percentage of the cases. The main statistical methods were Wilcoxon signed rank test, *χ* ^2^ test, and Mann–Whitney *U* test. In phase III, propensity score matching (PSM) was applied to reduce the effects of selection bias and potential confounding factors, in order to achieve balance or comparability of treatment groups (Glynn et al., 2006). The proportion of PSM was selected according to the proportion of original data between the case group and the control group, including 1:1, 1:2, and 1:3 (the remaining sample size after matching was affected by the difference of control variables between the groups). Combining with clinical prior knowledge, the controlling variables in PSM were determined as age, gender, length of hospital stay, duration of medication, ALT interval before treatment, ALT interval after treatment, baseline ALT level, surgery situation (non-surgery, hepatobiliary surgery, and nonhepatobiliary surgery), liver disease diagnosis (postoperation of tumor/liver transplantation, cirrhosis, hepatitis, abnormal liver function), and medical centers.

The indicators of economic evaluation mainly include total days of using PPC, hospitalization days, and total hospitalization cost. The total hospitalization cost includes the expense of medication, examination, and other expenses. A normally used economic analysis method in clinical, minimum cost analysis was performed for the medication groups without significant differences in ALT change level to compare the differences in total hospitalization cost between different treatment groups (*p* < 0.05 was considered significant) (Tam et al., 2017). The minimum cost refers to the analysis that compares the cost of different regimens in the case that there is no difference in the output or effect, benefit, and utility of the outcome, and the regimen with the minimum cost is given priority. Cost-effectiveness analysis was conducted for the medication groups with significant differences in ALT change level to compare the differences in cost-effectiveness ratio (C/E) among various medication groups. Cost-effectiveness analysis is the calculation of the cost of each unit of therapeutic effect of different regimens, measured against a specific clinical treatment purpose. Effectiveness was defined as a 50% decrease in ALT level after treatment or a change from abnormal (high/low) to normal ALT level after treatment. The C/E is the ratio of cost (C) to effect (E). The higher ratio indicates the higher cost of the regimen will be. However, the smallest C/E does not mean that it is the best regimen. When the cost increases, the corresponding effect will also increase, but not in a direct proportion. Normally, effect will increase with the augment of cost, but not proportionally. When cost increases to a certain amount, the increase in effect will gradually decrease or will no longer increase. When the cost and the effect increase at the same time, it is necessary to consider the cost of each additional effect unit, namely, the incremental cost-effectiveness ratio (△C/△E), which is the result of comparing other costs with the lowest cost as a reference. The lower ratio indicates the lower cost of adding one therapeutic effect, indicating greater practical significance of the regimen (Boersma et al., 2010). In addition, in order to better understand the uncertainty of this study, sensitivity analysis was used to confirm the stability of economic analysis result; we adjusted the economic evaluation data to a certain extent (medication costs were reduced by 10%; examination costs were increased by 5%; total efficiency decreased by 10%) to see if the results remained the same (Bowrin et al., 2020).

Age (years) = (date of admission − date of birth)/365.25, results rounded to a whole number. ALT/AST/TBil interval before treatment (days) = the start time of PPC injection or control drugs − the last ALT/AST/TBil examination time before treatment after admission. ALT/AST/TBil interval after treatment (days) = the last ALT/AST/TBil examination time after treatment before discharge − the start time of PPC injection or control drugs. Baseline ALT/AST/TBil was categorized as normal/low level and high level.

## Results

### Phase I

In the first phase of the study, a total of 44,069 cases of hospitalization records and 28,516 hospitalized patients were included (Table 1). Among the hospitalization records, 64.02% were male, median age was 59 years [IQR (interquartile range), 50–67] years, postoperation of tumor/liver transplantation ranked the first (38.09%) in the first clinical diagnosis. In liver disease spectrum, postoperation of tumor/liver transplantation, viral hepatitis, and liver cirrhosis ranked as the top three liver diseases, which occupied 38.09%, 21.23%, and 18.51%, respectively (Supplementary Table S1). The basic chronic diseases (hypertension, diabetes, and hyperlipidemia) accounted for 13.81%, 7.60%, and 0.29% of total hospitalization records, respectively. A total of 62.57% cases did not have surgery, 23.73% had surgery that may affect liver function, and 13.70% had surgery leading to nonsignificant/unknown effect on liver function. There were 9,889 cases (22.44%) that used PPC injection alone, followed by the combination of PPC + magnesium isoglycyrrhizinate (15.33%), PPC + glutathione (7.99%), and PPC + magnesium isoglycyrrhizinate + glutathione (5.26%). Glutathione and magnesium isoglycyrrhizinate were two of the most common drugs in combination with PPC for hepatoprotective therapy. Moreover, the median total days of using PPC were 6 days (IQR, 3–10 days), median hospitalization day was 9 days (IQR, 5–15 days), and the median total cost was 21,067 China yuan (CNY) (IQR, 11,893–42,763 CNY).

**TABLE 1:** Baseline information in phase Ⅰ

Categories	Multicenters
	Hospitalization records (N = 44,069)^a^
Gender (male), n (%)	28,214 (64.02)
Age,^b^ median (IQR), years	59 (50–67)
First clinical diagnosis,^c^ n (%)	
Postoperation of tumor/liver transplantation	16,784 (38.09)
Viral hepatitis	1,753 (3.98)
Liver cirrhosis	1,337 (3.03)
Space-occupying lesions/postoperative	1,154 (2.62)
Abnormal liver function	21,669 (49.17)
Drug-induced liver injury	182 (0.41)
Autoimmune liver disease	23 (0.05)
Alcoholic liver disease	18 (0.04)
Nonalcoholic fatty liver disease	437 (0.99)
Hepatic encephalopathy	51 (0.12)
Hepatic vascular diseases	49 (0.11)
Nonneoplastic diseases of the biliary tract	566 (1.28)
Others	46 (0.10)
Basic chronic disease, n (%)	
Hypertension	6,085 (13.81)
Diabetes	3,348 (7.60)
Hyperlipidemia	129 (0.29)
Surgery situation, n (%)	
Nonsurgery	27,574 (62.57)
Surgery may affect liver function (ALT)	10,459 (23.73)
No significant/unknown effect of surgery on liver function (ALT)	6,036 (13.70)
Medication, n (%)	
PPC injection	9,889 (22.44)
PPC injection + magnesium isoglycyrrhizinate	6,756 (15.33)
PPC injection + glutathione	3,519 (7.99)
PPC injection + glutathione + magnesium isoglycyrrhizinate	2,318 (5.26)
PPC injection + glutathione + ademetionine	1,305 (2.96)
PPC injection + acetylcysteine	1,146 (2.60)
PPC injection + magnesium isoglycyrrhizinate + ademetionine + glutathione	943 (2.14)
PPC injection + magnesium isoglycyrrhizinate + ademetionine + glutathione + acetylcysteine	756 (1.72)
PPC injection + ademetionine	733 (1.66)
PPC injection + compound glycyrrhizin	730 (1.66)
PPC injection + glutathione + acetylcysteine	694 (1.57)
PPC injection + glutathione + bicyclol	500 (1.13)
PPC injection + ademetionine + magnesium isoglycyrrhizinate	498 (1.13)
PPC injection + glutathione + magnesium isoglycyrrhizinate + compound glycyrrhizin	494 (1.12)
PPC injection + ademetionine + glutathione + acetylcysteine	468 (1.06)
Others	13,320 (30.24)
Total days of using PPC, median (IQR)	6 (3–10)
Hospitalization days, median (IQR)	9 (5–15)
Total treatment cost, median (IQR), CNY	21,067 (11,893–42,763)

a Hospitalization records were recorded according to the time of admission, and the record of multiple hospitalizations of one patient is not reprocessed.

b Age (years)= (admission date in the hospitalization record—date of birth in the patient’s information)/365.25.

c The first clinical diagnosis was based on discharge diagnosis.

Abbreviations: Min, minimum; Max, maximum; IQR, interquartile range; PPC, polyene phosphatidyl choline; ALT, alanine transaminase; CNY, China yuan.

### Phase II

In the second phase of the study, among 1,595 cases with PPC injection alone, five subgroups were included according to the liver disease spectrum, such as postoperation of tumor/liver transplantation, postoperation of non-tumor/liver transplantation, viral hepatitis, liver cirrhosis, and abnormal liver function group (Table 2). In the whole cohort (*n* = 1,595), 70.41% were male, median age was 60 years (IQR, 51–68 years), postoperation of tumor/liver transplantation ranked the first (48.78%) in the first clinical diagnosis. In liver disease spectrum, postoperation of tumor/liver transplantation, viral hepatitis, and liver cirrhosis ranked as the top three liver diseases, which occupied 48.78%, 27.27%, and 20.25%, respectively (Supplementary Table S1). The basic chronic diseases (hypertension, diabetes, and hyperlipidemia) accounted for 22.82%, 12.85%, and 0.94% of total hospitalization records, respectively. A total of 57.55% cases did not have surgery, 26.90% had surgery that may affect liver function, and 15.55% had surgery leading to nonsignificant/unknown effect on liver function. According to self-control results of ALT, AST, TBil recovery rate changes in PPC injection alone group in Table 3, ALT recovery ranged from 18.69% to 42.32%, AST recovery ranged from 17.59% to 59.20%, and TBil recovery ranged from 16.97% to 47.37%. In addition, ALT, AST, and TBil decreased significantly after PPC alone treatment in postoperation of non-tumor/liver transplantation group and abnormal liver function group, and ALT decreased significantly after PPC alone treatment in the whole group. Furthermore, the median total days of using PPC were 8 days (IQR, 5–12 days), median hospitalization day was 11 days (IQR, 8–17 days), and the median total cost was 27,925 CNY (IQR, 17,409–54,349 CNY).

**TABLE 2:** Baseline information in phase II

Categories	Multicenters					
	PPC (N = 1,595)	PPC1 (N = 778)	PPC2 (N = 817)	PPC3 (N = 435)	PPC4 (N = 323)	PPC5 (N = 478)
Gender (male), n (%)	1,123 (70.41%)	605 (77.76%)	518 (63.4%)	370 (85.06%)	260 (80.5%)	308 (64.44%)
Age,^b^ median (IQR), years	60 (51–68)	58 (50–66)	62 (52–72)	55 (47–63)	56 (49–66)	62 (53–73)
First clinical diagnosis,^c^ n (%)	—	—	—	—	—	—
Postoperation of tumor/liver transplantation	778 (48.78%)	778 (100.00%)	—	344 (79.08%)	236 (73.07%)	16 (3.35%)
Viral hepatitis	91 (5.71%)	—	91 (11.14%)	91 (20.92%)	26 (8.05%)	1 (0.21%)
Liver cirrhosis	61 (3.82%)	—	61 (7.47%)	—	61 (18.89%)	2 (0.42%)
Space-occupying lesions/postoperative	114 (7.15%)	—	114 (13.95%)	—	—	3 (0.63%)
Abnormal liver function	456 (28.59%)	—	456 (55.81%)	—	—	456 (95.40%)
Drug-induced liver injury	7 (0.44%)	—	7 (0.86%)	—	—	-
Autoimmune liver disease	—	—	—	—	—	-
Alcoholic liver disease	1 (0.06%)	—	1 (0.12%)	—	—	—
Nonalcoholic fatty liver disease	46 (2.88%)	—	46 (5.63%)	—	—	—
Hepatic encephalopathy	1 (0.06%)	—	1 (0.12%)	—	—	—
Hepatic vascular diseases	4 (0.25%)	—	4 (0.49%)	—	—	—
Nonneoplastic diseases of the biliary tract	25 (1.57%)	—	25 (3.06%)	—	—	—
Others	11 (0.69%)	—	11 (1.35%)	—	—	—
Basic chronic disease, n (%)		—	—	—	—	—
Hypertension	364 (22.82)	101 (12.98)	263 (32.19)	66 (15.17)	45 (13.93)	178 (37.24)
Diabetes	205 (12.85)	53 (6.81)	152 (18.60)	33 (7.59)	32 (9.91)	93 (19.46)
Hyperlipidemia	15 (0.94)	1 (0.13)	14 (1.71)	1 (0.23)	0 (0.00)	6 (1.26)
Surgery situation, n (%)	—	—	—	—	—	—
Nonsurgery	918 (57.55)	342 (43.96)	576 (70.5)	202 (46.44)	148 (45.82)	364 (76.15)
Surgery may affect liver function (ALT)	429 (26.9)	331 (42.54)	98 (12.0)	196 (45.06)	132 (40.87)	39 (8.16)
No significant/unknown effect of surgery on liver	248 (15.55)	105 (13.5)	143 (17.5)	37 (8.51)	43 (13.31)	75 (15.69)
Function(ALT)						
Total days of using PPC, days, median (IQR)	8 (5–12)	8 (5–12)	8 (5–12)	8 (5–12)	9 (6–13)	8 (6–12)
Hospitalization days, days, median (IQR)	11 (8–17)	10 (8–15)	13 (8–20)	11 (8–14)	11 (8–15)	13 (9–20)
Total treatment cost, CNY, median (IQR)	27,925 (17,409–54,349)	23,525 (16,707–42,766)	35,012 (18,152–63,819)	23,768 (16,417–42,885)	23,047 (16,124–39,189)	45,546 (22,266–72,113)

Notes: PPC1 indicates using PPC injection alone and liver disease spectrum is “postoperation of tumor/liver transplantation”; PPC2 indicates using PPC injection alone and liver disease spectrum is “postoperation of nontumor/liver transplantation”; PPC3 indicates using PPC injection alone and liver disease spectrum is “viral hepatitis”; PPC4 indicates using PPC injection alone and liver disease spectrum is “liver cirrhosis”; PPC5 indicates using PPC injection alone and liver disease spectrum is “abnormal liver function”.

a Hospitalization records were recorded according to the time of admission, and the record of multiple hospitalizations of one patient is not reprocessed.

b Age (years) = (admission date in the hospitalization record—date of birth in the patient’s information)/365.25.

c The first clinical diagnosis was based on discharge diagnosis.

Abbreviations: Min, minimum; Max, maximum; IQR, interquartile range; PPC, polyene phosphatidyl choline; CNY, China yuan; ALT, alanine transaminase.

**TABLE 3:** Changes of ALT/AST/TBil after PPC injection.

Categories	Multicenters					
	PPC (N = 1,595)	PPC1 (N = 778)	PPC2 (N= 817)	PPC3 (N = 435)	PPC4 (N = 323)	PPC5 (N = 478)
ALT recovery, n (%)	277 (35.79)	40 (18.69)	237 (42.32)	27 (20.77)	16 (20.78)	175 (39.24)
ALT change						
Median (IQR)	−1 (−22–21)	8 (−3–38)	−12 (−40–3)	7 (−5–45)	2 (−5–29)	−26 (−56–1)
Wilcoxon signed rank test, *p* value	−4.6 (0.000)	−11.4 (0.000)	−16.5 (0.000)	−7.6 (0.000)	−5.5 (0.000)	−14.7 (0.000)
AST recovery, n (%)	302 (42.30)	51 (17.59)	251 (59.20)	40 (22.47)	24 (20.69)	186 (58.68)
AST change						
Median (IQR)	0 (−17–26)	10 (−3–51)	−8 (−48–6)	9 (−4–64)	4 (−5–42)	−23 (−95–4)
Wilcoxon signed rank test, *p* value	−8.8 (0.000)	−14.4 (0.000)	−18.6 (0.000)	−8.9 (0.000)	−7.3 (0.000)	−16.6 (0.000)
TBil recovery, n (%)	184 (30.62)	53 (17.04)	131 (45.17)	39 (21.91)	28 (16.97)	81 (47.37)
TBil change						
Median (IQR)	0 (−4–7)	4 (0–12)	−2 (−8–2)	4 (−1–12)	3 (−2–11)	−2 (−10–1)
Wilcoxon signed rank test, *p* value	−8.3 (0.000)	−13.5 (0.000)	−9.6 (0.000)	−9.8 (0.000)	−7.4 (0.000)	−11.1 (0.000)

Notes: PPC1 indicates using PPC injection alone and liver disease spectrum is “postoperation of tumor/liver transplantation”; PPC2 indicates using PPC injection alone, and liver disease spectrum is “postoperation of nontumor/liver transplantation”; PPC3 indicates using PPC injection alone, and liver disease spectrum is “viral hepatitis”; PPC4 indicates using PPC injection alone, and liver disease spectrum is “liver cirrhosis”; PPC5 indicates using PPC injection alone, and liver disease spectrum is “abnormal liver function”. ALT change indicates ALT level relative to baseline after treatment; ALT recovery indicates cases with abnormal ALT that changes to normal range (≤40 U/L) after treatment; AST change indicates AST level relative to baseline after treatment; AST recovery indicates cases with abnormal AST that changes to normal range (≤40 U/L) after treatment; TBil change indicates TBil level relative to baseline after treatment; TBil recovery indicates cases with abnormal TBil that changes to normal range (≤17.1 μmol/L) after treatment.

Abbreviations: IQR, interquartile range; PPC, polyene phosphatidyl choline; ALT, alanine transaminase; AST, aspartate aminotransferase; TBil, total bilirubin.

### Phase III

The baseline information in the third phase is illustrated in Supplementary Tables S2, S7; case–control groups were included, such as PPC injection alone (*n* = 1,595), magnesium isoglycyrrhizinate injection alone (*n* = 3,300), glutathione injection alone (*n* = 3,188), PPC + glutathione injection (*n* = 1,517), PPC + magnesium isoglycyrrhizinate injection (*n* = 1,331), PPC + glutathione + magnesium isoglycyrrhizinate injection (*n* = 1,102), and glutathione + magnesium isoglycyrrhizinate injection (*n* = 4,013). Among all seven groups, postoperation of tumor/liver transplantation occupied the highest proportion in the first clinical diagnosis and the liver disease spectrum.

Table 4 shows the ALT change value and test results, which indicate significant decrease (*p* < 0.05) in different medication combinations after PSM, and AST and TBil results are displayed in Supplementary Tables S3, S4. Specifically, in the whole group, the decrease in ALT was significantly higher after treatment with PPC alone than glutathione alone (*p* = 0.045), and the decrease in ALT was significantly higher after treatment with PPC + magnesium isoglycyrrhizinate than magnesium isoglycyrrhizinate alone (*p* = 0.000). Therefore, for the whole group, PPC alone was more effective than glutathione alone, and PPC + magnesium isoglycyrrhizate was more effective than magnesium isoglycyrrhizate alone. In the postoperation of non-tumor/liver transplantation group, the decrease in ALT was significantly higher after treatment with PPC alone than glutathione alone (*p* = 0.002), the decrease in ALT after PPC + magnesium isoglycyrrhizate treatment was significantly higher than that after magnesium isoglycyrrhizate alone (*p* = 0.000), the decrease in ALT after PPC + glutathione treatment was significantly higher than that after glutathione alone (*p* = 0.020), the decrease in ALT after PPC + magnesium isoglycyrrhizate treatment was significantly higher than that after glutathione + magnesium isoglycyrrhizate (*p* = 0.000), and the decrease in ALT after PPC + magnesium isoglycyrrhizate + glutathione treatment was significantly higher than that after magnesium isoglycyrrhizate + glutathione (*p* = 0.039). In a word, for postoperation of non-tumor/liver transplantation group, the effectiveness of PPC alone was superior to that of glutathione alone, the effectiveness of PPC + glutathione was superior to that of glutathione alone, the effectiveness of PPC + magnesium isoglycyrrhizate was superior to that of magnesium isoglycyrrhizate alone and glutathione + magnesium isoglycyrrhizate, and the effectiveness of PPC + magnesium isoglycyrrhizinate + glutathione was superior to that of magnesium isoglycyrrhizinate + glutathione. In the abnormal liver function group, the decrease in ALT was significantly higher after treatment with PPC + magnesium isoglycyrrhizate than magnesium isoglycyrrhizate alone (*p* = 0.043) and glutathione + magnesium isoglycyrrhizate (*p* = 0.000), the decrease in ALT after PPC + glutathione treatment was significantly higher than that after magnesium isoglycyrrhizate + glutathione (*p* = 0.021), and the decrease in ALT after PPC + magnesium isoglycyrrhizate + glutathione treatment was significantly higher than that after magnesium isoglycyrrhizate + glutathione (*p* = 0.006). We can see that for the abnormal liver function group, the effectiveness of PPC + magnesium isoglycyrrhizate was superior to that of magnesium isoglycyrrhizate alone and glutathione + magnesium isoglycyrrhizate, the effectiveness of PPC + glutathione was superior to that of magnesium isoglycyrrhizate + glutathione, and the effectiveness of PPC + magnesium isoglycyrrhizinate + glutathione was superior to that of magnesium isoglycyrrhizinate + glutathione. Lastly, for postoperation of tumor/liver transplantation group, viral hepatitis group, and liver cirrhosis group, the subgroups using PPC alone or combination could not significantly decrease ALT level. The results of AST and TBil in all groups were basically consistent with the ALT result. Nevertheless, for all subgroups, in terms of ALT recovery, AST recovery, and TBil recovery, the increase was not remarkable.

**TABLE 4:** ALT change value and test results in different medication combinations.

No	Medication combination	Sample size before PSM, N	Sample size after PSM, N	ALT recovery		ALT change	
				n (%)	*χ* ^2^ Test (*p* value)	Median	Mann–Whitney *U* test (*p* value)
**The whole group**							
1	Glutathione	3,300	2,823	411 (34.51%)	0.4 (0.545)	0	2,164,248.5 (0.045)
	PPC	1,595	1,591	276 (35.84%)		−1	
2	Magnesium isoglycyrrhizinate	3,188	2,299	383 (35.36%)	0.03 (0.873)	0	1,746,021.5 (0.756)
	PPC	1,595	1,528	259 (35.00%)		−1	
3	Magnesium isoglycyrrhizinate	3,188	1,828	358 (35.13%)	0.5 (0.490)	−3	1,279,641.0 (0.000)
	PPC + magnesium isoglycyrrhizinate	1,331	1,296	269 (33.58%)		−7	
4	Glutathione	3,300	2,222	340 (32.17%)	0.1 (0.736)	−1	1,670,468.0 (0.852)
	PPC + glutathione	1,517	1,509	259 (32.91%)		−1	
5	Magnesium isoglycyrrhizinate + glutathione	4,013	2,473	433 (29.60%)	2.3 (0.128)	−5	1,716,065.5 (0.000)
	PPC + glutathione	1,517	1,503	257 (32.70%)		−1	
6	Glutathione + magnesium isoglycyrrhizinate	4,013	2,160	375 (30.89%)	0.3 (0.598)	−2	1,067,183.0 (0.377)
	PPC + magnesium isoglycyrrhizinate	1,331	969	186 (32.12%)		−3	
7	Magnesium isoglycyrrhizinate + glutathione	4,013	2,658	542 (32.30%)	0.6 (0.447)	−2	1,447,937.0 (0.947)
	PPC + magnesium isoglycyrrhizinate + glutathione	1,102	1,088	240 (33.90%)		−5	
**Postoperation of tumor/liver transplantation group**							
1	Glutathione	1,155	746	45 (21.74%)	1.0 (0.312)	5	250,536.5 (0.001)
	PPC	778	746	37 (17.79%)		9	
2	Magnesium isoglycyrrhizinate	1,296	678	45 (22.73%)	2.3 (0.126)	5	210,321.5 (0.007)
	PPC	778	678	32 (16.58%)		10	
3	Magnesium isoglycyrrhizinate	1,296	761	59 (22.52%)	0.1 (0.811)	4	213,449.0 (0.308)
	PPC + magnesium isoglycyrrhizinate	574	543	46 (23.47%)		4	
4	Glutathione	1,155	844	81 (22.88%)	0.03 (0.873)	1	348,908.0 (0.468)
	PPC + glutathione	874	844	80 (23.39%)		2	
5	Magnesium isoglycyrrhizinate + glutathione	1,821	1,415	153 (22.70%)	0.1 (0.710)	−1	559,381.5 (0.000)
	PPC + glutathione	874	873	84 (23.73%)		2	
6	Glutathione + magnesium isoglycyrrhizinate	1,821	1,189	96 (21.15%)	0.3 (0.587)	2	238,756.5 (0.012)
	PPC + magnesium isoglycyrrhizinate	574	437	31 (19.14%)		6	
7	Magnesium isoglycyrrhizinate + glutathione	1,821	1,300	140 (23.06%)	1.3 (0.257)	−1	321,130.5 (0.064)
	PPC + magnesium isoglycyrrhizinate + glutathione	554	523	65 (26.75%)		0	
**Postoperation of nontumor/liver transplantation group**							
1	Glutathione	2,145	1,260	300 (38.81%)	1.3 (0.256)	−4	552,529.0 (0.002)
	PPC	817	813	233 (41.91%)		−12	
2	Magnesium isoglycyrrhizinate	1,892	1,208	302 (38.97%)	1.2 (0.270)	−7	481,863.0 (0.120)
	PPC	817	766	219 (42.03%)		−11	
3	Magnesium isoglycyrrhizinate	1,892	1,000	292 (39.67%)	1.6 (0.207)	−18	400,708.5 (0.000)
	PPC + magnesium isoglycyrrhizinate	757	720	210 (36.27%)		−28	
4	Glutathione	2,145	1,291	304 (39.07%)	0.02 (0.883)	−3	421,744.0 (0.020)
	PPC + glutathione	643	613	162 (39.51%)		−8	
5	Magnesium isoglycyrrhizinate + glutathione	2,192	1,181	330 (38.19%)	0.4 (0.541)	−11	370,871.0 (0.733)
	PPC + glutathione	643	622	171 (39.95%)		−10	
6	Glutathione + magnesium isoglycyrrhizinate	2,192	969	290 (37.91%)	0.2 (0.635)	−9	284,621.5 (0.000)
	PPC + magnesium isoglycyrrhizinate	757	517	162 (39.32%)		−23	
7	Magnesium isoglycyrrhizinate + glutathione	2,192	1,284	378 (36.10%)	0.9 (0.336)	−3	366,547.5 (0.039)
	PPC + magnesium isoglycyrrhizinate + glutathione	548	538	175 (38.72%)		−14	
**Abnormal liver function group**							
1	Glutathione	638	543	196 (40.41%)	0.7 (0.388)	−21	114,423.5 (0.122)
	PPC	478	398	138 (37.50%)		−25	
2	Magnesium isoglycyrrhizinate	879	409	150 (39.58%)	0.3 (0.575)	−31	78,373.0 (0.119)
	PPC	478	409	144 (37.60%)		−26	
3	Magnesium isoglycyrrhizinate	879	470	173 (39.14%)	1.7 (0.192)	−34	118,865.0 (0.043)
	PPC + magnesium isoglycyrrhizinate	501	470	155 (34.91%)		−41	
4	Glutathione	638	546	191 (39.22%)	0.3 (0.597)	−18	100,221.5 (0.091)
	PPC + glutathione	376	344	112 (37.33%)		−26	
5	Magnesium isoglycyrrhizinate + glutathione	1,195	730	245 (37.01%)	0.2 (0.698)	−17	142,652.5 (0.021)
	PPC + glutathione	376	360	121 (38.29%)		−28	
6	Glutathione + magnesium isoglycyrrhizinate	1,195	511	168 (35.74%)	0.1 (0.814)	−17	102,217.5 (0.000)
	PPC + magnesium isoglycyrrhizinate	501	348	117 (36.56%)		−34	
7	Magnesium isoglycyrrhizinate + glutathione	1,195	800	261 (35.13%)	0.6 (0.425)	6	158,820.0 (0.006)
	PPC + magnesium isoglycyrrhizinate + glutathione	372	361	111 (32.65%)		−15	
**Viral hepatitis group**							
1	Glutathione	797	638	51 (21.98%)	0.2 (0.685)	4	125,857.0 (0.011)
	PPC	435	434	26 (20.16%)		7	
2	Magnesium isoglycyrrhizinate	927	577	28 (16.87%)	0.3 (0.602)	8	111,015.0 (0.725)
	PPC	435	390	22 (19.30%)		8	
3	Magnesium isoglycyrrhizinate	927	421	29 (20.14%)	0.4 (0.517)	6	65,093.5 (0.030)
	PPC + magnesium isoglycyrrhizinate	294	282	27 (23.48%)		2	
4	Glutathione	797	487	38 (17.84%)	0.7 (0.390)	4	119,145.5 (0.898)
	PPC + glutathione	567	487	43 (21.18%)		4	
5	Magnesium isoglycyrrhizinate + glutathione	1,266	872	86 (21.39%)	0.1 (0.756)	2	240,491.5 (0.414)
	PPC + glutathione	567	566	53 (20.38%)		3	
6	Glutathione + magnesium isoglycyrrhizinate	1,266	735	68 (22.90%)	0.2 (0.639)	3	93,569.5 (0.619)
	PPC + magnesium isoglycyrrhizinate	294	260	23 (20.72%)		4	
7	Magnesium isoglycyrrhizinate + glutathione	1,266	823	92 (23.29%)	0.05 (0.830)	2	129,848.5 (0.970)
	PPC + magnesium isoglycyrrhizinate + glutathione	318	316	35 (22.44%)		2	
**Liver cirrhosis group**							
1	Glutathione	486	314	18 (23.08%)	0.7 (0.399)	3	49,657.5 (0.874)
	PPC	323	314	13 (17.57%)		2	
2	Magnesium isoglycyrrhizinate	590	265	25 (29.76%)	1.8 (0.181)	7	34,043.5 (0.544)
	PPC	323	265	14 (20.29)		4	
3	Magnesium isoglycyrrhizinate	590	333	31 (28.97%)	0.1 (0.815)	7	46,345.0 (0.030)
	PPC + magnesium isoglycyrrhizinate	279	252	25 (27.47%)		1	
4	Glutathione	486	366	27 (20.45%)	0.3 (0.609)	2	69,325.5 (0.412)
	PPC + glutathione	463	366	32 (23.02%)		2	
5	Magnesium isoglycyrrhizinate + glutathione	896	683	67 (23.34%)	0.1 (0.744)	2	159,178.0 (0.798)
	PPC + glutathione	463	462	39 (22.03%)		1	
6	Glutathione + magnesium isoglycyrrhizinate	896	589	54 (25.12%)	0.02 (0.897)	4	65,596.5 (0.863)
	PPC + magnesium isoglycyrrhizinate	279	221	20 (24.39%)		3	
7	Magnesium isoglycyrrhizinate + glutathione	896	608	66 (27.27%)	0.03 (0.860)	2	82,097.0 (0.366)
	PPC + magnesium isoglycyrrhizinate + glutathione	260	260	31 (28.18%)		0	

Notes: The data size of some medication combinations in the nontumor-abnormal liver function group was too small to be included in the analysis. ALT change indicates ALT level relative to baseline after treatment; ALT recovery indicates cases with abnormal ALT that changes to normal range (≤40 U/L) after treatment.

Abbreviations: ALT, alanine transaminase; PSM, propensity score matching; PPC, polyene phosphatidyl choline.

In the economic analysis, because of the small sample size or the lack of relevant data of other groups, only the whole group, postoperation of non-tumor/liver transplantation group, and abnormal liver function group were included. In terms of total hospitalization costs and C/E, drug alone or combination that had less total hospitalization cost (*p* < 0.05) and smaller C/E was proven to be more effective. In the whole group and postoperation of non-tumor/liver transplantation group, PPC alone was more economical than glutathione alone, PPC + magnesium isoglycyrrhizinate was more economical than magnesium isoglycyrrhizinate alone and magnesium isoglycyrrhizinate + glutathione, and PPC + glutathione was more economical than magnesium isoglycyrrhizinate + glutathione (Tables 5 and 6). In the abnormal liver function group, PPC + magnesium isoglycyrrhizinate was more economical than magnesium isoglycyrrhizinate alone and magnesium isoglycyrrhizinate + glutathione, and PPC + glutathione and PPC + glutathione + magnesium isoglycyrrhizinate were more economical than magnesium isoglycyrrhizinate + glutathione (Tables 5, 6). The results of sensitivity analysis were consistent with the economic analysis (Supplementary Tables S5, S6).

**TABLE 5:** Cost minimization analysis in phase Ⅲ

No	Medication combination	Hospitalization records (N)	Total hospitalization costs (mean), CNY	*p* value
**The whole group**				
1	PPC	1,528	31,488.6	0.125
	Magnesium isoglycyrrhizinate	2,299	33,933.8	
2	PPC + glutathione	1,509	40,797.1	0.000
	Glutathione	2,222	34,985.2	
3	PPC + magnesium isoglycyrrhizinate	969	35,962.3	0.000
	Magnesium isoglycyrrhizinate + glutathione	2,160	42,547.3	
4	PPC + magnesium isoglycyrrhizinate + glutathione	1,088	44,362.7	0.964
	Magnesium isoglycyrrhizinate + glutathione	2,658	44,864.0	
**Postoperation of nontumor/liver transplantation group**				
1	PPC	766	33,965.5	0.172
	Magnesium isoglycyrrhizinate	1,208	37,730.1	
2	PPC + glutathione	622	43,073.6	0.215
	Magnesium isoglycyrrhizinate + glutathione	1,181	47,122.2	
**Abnormal liver function group**				
1	PPC	398	40,018.9	0.708
	Glutathione	543	47,284.4	
2	PPC	409	35,925.6	0.101
	Magnesium isoglycyrrhizinate	409	42,245.6	
3	PPC + glutathione	344	49,194.6	0.000
	Glutathione	546	42,243.8	

Abbreviations: PPC, polyene phosphatidyl choline; CNY, China yuan.

**TABLE 6:** Cost-effectiveness analysis in phase Ⅲ

No	Medication combination	Hospitalization records (N)	Effective records (N)	Costs (CNY)	Total effective rate (effectiveness, E%)	Cost-effectiveness ratio (C/E)	Incremental cost-effectiveness ratio (△C/△E)
**The whole group**							
1	PPC	1,591	471	35,848.4	29.60	121,092.9	35,818.6
	Glutathione	2,823	778	35,117.7	27.56	127,425.9	
2	PPC + magnesium isoglycyrrhizinate	1,296	510	30,912.8	39.35	78,555.0	−9,713.9
	Magnesium isoglycyrrhizinate	1,828	651	31,276.1	35.61	87,823.0	
3	PPC + glutathione	1,503	586	39,260.8	38.99	100,698.0	−153,102.6
	Magnesium isoglycyrrhizinate + glutathione	2,473	945	40,455.0	38.21	105,867.9	
**Postoperation of nontumor/liver transplantation group**							
1	PPC	813	393	37,966.7	48.34	78,541.7	−24,507.2
	Glutathione	1,260	504	40,010.6	40.00	100,026.5	
2	PPC + magnesium isoglycyrrhizinate	720	389	33,103.7	54.03	61,271.6	−49,406.1
	Magnesium isoglycyrrhizinate	1,000	511	34,551.3	51.10	67,615.0	
3	PPC + glutathione	613	273	43,619.9	44.54	97,945.1	66,834.5
	Glutathione	1,291	504	39,944.0	39.04	102,316.8	
4	PPC + magnesium isoglycyrrhizinate	517	276	38,730.8	53.38	72,550.1	−210,830.3
	Magnesium isoglycyrrhizinate + glutathione	969	491	44,444.3	50.67	87,711.8	
5	PPC + magnesium isoglycyrrhizinate + glutathione	538	287	53,162.5	53.35	99,656.6	101,440.0
	Magnesium isoglycyrrhizinate + glutathione	1,284	669	51,894.5	52.10	99,600.1	
**Abnormal liver function group**							
1	PPC + magnesium isoglycyrrhizinate	470	283	31,842.6	60.21	52,883.4	188,085.5
	Magnesium isoglycyrrhizinate	470	294	36,243.8	62.55	57,940.8	
2	PPC + glutathione	360	200	48,161.3	55.56	86,690.3	186,462.4
	Magnesium isoglycyrrhizinate + glutathione	730	425	53,121.2	58.22	91,243.6	
3	PPC + magnesium isoglycyrrhizinate	348	208	37,816.5	59.77	63,269.9	−435,110.3
	Magnesium isoglycyrrhizinate + glutathione	511	292	49,259.9	57.14	86,204.9	
4	PPC + magnesium isoglycyrrhizinate + glutathione	361	188	48,996.1	52.08	94,082.9	189,558.8
	Magnesium isoglycyrrhizinate + glutathione	800	433	52,863.1	54.12	97,668.6	

Abbreviations: PPC, polyene phosphatidyl choline; CNY, China yuan; C, cost; E, effectiveness.

## Discussion

Recently, PPC has been widely used in the treatment of liver disease. However, the effectiveness and economic evaluation of PPC on liver disease have not been comprehensively explained. This study had three phases, which were descriptive study of patients using PPC injection, self-control case study of patients using PPC injection, and case–control study among specific-disease groups using PPC injection or combination drugs, respectively. Findings in phase I indicate that glutathione and magnesium isoglycyrrhizinate were two of the most common drugs in combination with PPC for hepatoprotective therapy. The main findings in phase II (self-control case study) indicate that in the whole group, postoperation of non-tumor/liver transplantation group, and abnormal liver function group, ALT decreased significantly after treatment with PPC injection alone. The main findings in phase III (case–control study) indicate that PPC alone or in combination was more effective than glutathione alone or magnesium isoglycyrrhizate alone or their combination, especially in patients with postoperation of non-tumor/liver transplantation and/or abnormal liver function. Moreover, PPC shows better economic advantage, either used alone or in combination with glutathione and magnesium isoglycyrrhizinate.

Results in phase I indicate that PPC injection was commonly used among patients with liver disease, mainly those with postoperation of tumor/liver transplantation, viral hepatitis, and liver cirrhosis, and glutathione and magnesium isoglycyrrhizinate were two of the most common drugs in combination with PPC for hepatoprotective treatment. To date, multiple studies have investigated the mechanisms of PPC in treating liver diseases. PPC, as the main active component of human essential phospholipids, has high bioavailability and affinity for cell membranes and can repair cell membranes *via* maintaining the integrity and function of biofilm (Feng et al., 20202020). This is thought to help PPC repair damaged liver cell membranes in patients with liver injury, viral hepatitis, or nonalcoholic steatohepatitis (Cao et al., 2016). In addition, studies on NAFLD and nonalcoholic steatohepatitis have found that PPC can inhibit inflammatory factors and nuclear factor κB signaling pathway and regulate oxidative balance, indicating the therapeutic role of PPC on liver disease (Cao et al., 2016; Wang and Chen, 2016). Furthermore, a study of alcoholic liver disease showed that PPC has anti-inflammatory, antiapoptotic, antifibrotic, and antioxidant effects on alcoholic liver disease (Ikeda et al., 2011; Ma et al., 2011). Specifically, PPC contributes to inhibiting the overexpression of reactive oxygen species–generating enzymes to reduce ethanol-derived oxidative stress and inhibiting the expression of transforming growth factor β1 and activation of hepatic stellate cells to delay the occurrence of hepatic fibrosis (Ikeda et al., 2011). In addition, the down-regulation of nonphagocytic oxidase-4 is thought to be one of the antiapoptotic mechanisms of PPC (Ikeda et al., 2011). These were consistent with the results of the study on the protective effect of PPC on tissue injury induced by radiotherapy, that PPC may prevent cell death via regulating tissue activities of antioxidant enzymes (Zhang et al., 2019). In terms of the effect of PPC on lipid metabolism, it has been proven that PPC treatment can help liver absorb external free fatty acids, inhibit the expression of fatty acid transporter, and improve liver fatty acid metabolism (Yu et al., 2019). Moreover, as platinum-based chemotherapy often induces hepatoxicity, PPC can be used as a liver protective nutritional supplement for tumor therapy (Zhang et al., 2019).

We showed in phase II that PPC treatment was effective in the whole group, postoperation of non-tumor/liver transplantation group, and abnormal liver function group (ALT/AST/TBil level and recovery rate decreased significantly after PPC treatment; *p* < 0.05). According to the consensus on the treatment with PPC in patients with liver diseases, PPC was recommended for NAFLD patients with elevated ALT, AST, and γ-glutamyltransferase (GGT) after 3 months of basic therapy, patients with alcoholic liver disease who had recurrent abnormal liver function after basic therapy, and chronic hepatitis B patients with abnormal liver enzymes, and PPC can be used as adjuvant treatment for patients with moderate to severe drug-induced liver injury whose liver function damage continued to progress (Committee of the treatmen, 2017). One study about the effect of PPC in non–small cell lung cancer (NSCLC) patients demonstrated the protective impact of daily PPC administration on radiation-induced tissue injury and suggested PPC as adjuvant in NSCLC patients receiving radiation therapy (Zhang et al., 2019). Patients who received a complete course of PPC had a significantly lower risk of developing radiation pneumonitis than those without PPC supplementation (27.6% vs 43.5%) (Zhang et al., 2019). A real-world research of 2,843 adult patients having NAFLD with metabolic comorbidities from Russia revealed that PPC could consistently decrease AST, ALT, and GGT levels (*p* < 0.001), and another meta-analysis also showed that PPC was effective in lowering AST, ALT, and TBil levels (*p* < 0.01) (Maev et al., 2020; Luo et al., 2021).

In phase III, for drug effectiveness, the combination of PPC was more effective than glutathione alone or magnesium isoglycyrrhizate alone or their combination based on the change in ALT/AST/TBil levels. However, in this study, the recovery rate of ALT/AST/TBil shows no remarkable increase. Future research with more samples could refine the results. Previous studies have proven the effects of glutathione and magnesium isoglycyrrhizinate treatment alone. The antioxidant and detoxification abilities of glutathione alleviate hepatocyte edema and steatosis and inhibits ALT and AST elevation (Locigno and Castronovo, 2001; Lv et al., 2019; Vairetti et al., 2021). A meta-analysis focusing on efficacy and safety of PPC combined with glutathione proved that the combined drugs could significantly reduce ALT/AST/TBil compared with a single use of PPC or glutathione (*p* < 0.05) (Shi et al., 2018). Magnesium isoglycyrrhizinate is a derivative of an active component of *Glycyrrhiza glabra*, which is widely used for the treatment of liver fibrosis and inflammatory liver diseases because of its anti-inflammatory and hepatoprotective effects (Xie et al., 2015; Sui et al., 2018; Jiang et al., 2020). A study on the efficacy of PPC and magnesium isoglycyrrhizinate in treating drug-induced liver injury found that 34.97% of patients in both treatments achieved normal ALT levels (Lei et al., 2021). Herein, we believed that PPC, glutathione, and magnesium isoglycyrrhizinate have synergistic effects, which enhance the stabilization of liver cell membrane, antioxidant, detoxification, and anti-inflammatory effects. Few previous studies focused on evaluating PPC economy; our study provided plenty and valuable information for reference in clinical settings.

To our knowledge, this study is the first study to investigate the effectiveness and economy of PPC in patients with liver diseases based on real-world evidence from multicenters. We described the current usage of PPC in Chinese hospital and compared the effectiveness and economy of using liver-protective drugs alone or combination medication in different subgroups.

One advantage is that we comprehensively evaluated the economy of PPC by using different methods. The minimum cost analysis and cost-effectiveness analysis show the economic advantage of PPC than glutathione and magnesium isoglycyrrhizinate, and the sensitivity analysis confirms the credibility and stability of economic analysis result. In addition, various drug combinations were compared among different subgroups to sufficiently evaluate the application of PPC. One limitation to this study is the lack of drug safety data, such as adverse drug effect, which should be investigated in the future to complete a comprehensive evaluation of PPC usage. Another limitation is the unremarkable increase in ALT/AST/TBil recovery rate, we used only ALT/AST/TBil change rates with significance to show remarkable liver function improvement, which needs more samples in future research to validate the results.

## Conclusion

We found that PPC was effective and economical in protecting liver function in patients with postoperation of nontumor/liver transplantation and abnormal liver function, and one noteworthy outcome was that PPC could enhance the liver protective function of glutathione and magnesium isoglycyrrhizinate. In the future, we will endeavor to include more clinical data to expand the database and improve the quality of results.

## Data Availability

The raw data supporting the conclusion of this article will be made available by the authors, without undue reservation.
