# Supplementary material for: Effectiveness and Economic Evaluation of Polyene Phosphatidyl Choline in Patients With Liver Diseases Based on Real-World Research
Source: Front Pharmacol. 2022 Mar 7;13:806787. doi: 10.3389/fphar.2022.806787 (PMC8940240; doi:10.3389/fphar.2022.806787)
Supplement: Supplementary file 1 [file Table1.DOCX]

Supplementary Table S1. Liver disease spectrum **in phase Ⅰ and Ⅱ**

| **Categories** | **Phase Ⅰ** | **Phase Ⅱ** | | | | | |
| --- | --- | --- | --- | --- | --- | --- | --- |
|  | **Hospitalization records ^b^ (N=44069)** | **PPC**  **(N=1595)** | **PPC1**  **(N=778)** | **PPC2**  **(N=817)** | **PPC3**  **(N=435)** | **PPC4**  **(N=323)** | **PPC5**  **(N=478)** |
| Postoperation of tumor / liver transplantation | 16784(38.09%) | 778  (48.78%) | 778  (100.00%) | 0  (0.00%) | 344  (79.08%) | 236  (73.07%) | 16  (3.35%) |
| Viral hepatitis | 9357(21.23%) | 435  (27.27%) | 344  (44.22%) | 91  (11.14%) | 435  (100.00%) | 211  (65.33%) | 5  (1.05%) |
| Liver cirrhosis | 8158(18.51%) | 323  (20.25%) | 236  (30.33%) | 87  (10.65%) | 211  (48.51%) | 323  (100.00%) | 4  (0.84%) |
| Space-occupying lesions/postoperative | 4148(9.41%) | 222  (13.92%) | 79  (10.15%) | 143  (17.50%) | 40  (9.20%) | 29  (8.98%) | 7  (1.46%) |
| Abnormal liver function | 2526(5.73%) | 478  (29.97%) | 16  (2.06%) | 462  (56.55%) | 5  (1.15%) | 4  (1.24%) | 478  (100.00%) |
| Drug-induced liver injury | 340(0.77%) | 7(0.44%) | 0(0.00%) | 7(0.86%) | 0(0.00%) | 0(0.00%) | 0(0.00%) |
| Autoimmune liver disease | 98(0.22%) | 0(0.00%) | 0(0.00%) | 0(0.00%) | 0(0.00%) | 0(0.00%) | 0(0.00%) |
| Alcoholic liver disease | 76(0.17%) | 3(0.19%) | 1(0.13%) | 2(0.24%) | 1(0.23%) | 1(0.31%) | 0(0.00%) |
| Non alcoholic fatty liver disease | 805(1.83%) | 59(3.70%) | 1(0.13%) | 58(7.10%) | 3(0.69%) | 1(0.31%) | 5(1.05%) |
| Hepatic encephalopathy | 154(0.35%) | 3(0.19%) | 0(0.00%) | 3(0.37%) | 1(0.23%) | 2(0.62%) | 0(0.00%) |
| Hepatic vascular diseases | 107(0.24%) | 6(0.38%) | 1(0.13%) | 5(0.61%) | 1(0.23%) | 1(0.31%) | 0(0.00%) |
| Non neoplastic diseases of the biliary tract | 1005(2.28%) | 40(2.51%) | 10(1.29%) | 30(3.67%) | 1(0.23%) | 4(1.24%) | 1(0.21%) |
| Others | 355(0.81%) | 11(0.69%) | - | 11(1.35%) | - | - | - |

Notes: PPC1 indicates using PPC injection alone and liver disease spectrum is “postoperation of tumor / liver transplantation”; PPC2 indicates using PPC injection alone and liver disease spectrum is “postoperation of non-tumor / liver transplantation”; PPC3 indicates using PPC injection alone and liver disease spectrum is “viral hepatitis”; PPC4 indicates using PPC injection alone and liver disease spectrum is “liver cirrhosis”; PPC5 indicates using PPC injection alone and liver disease spectrum is “abnormal liver function”.

^a^: Liver disease spectrum was recorded according to the discharge diagnosis in the hospitalization records (extracting all diagnoses related to the word "hepatic" or "liver").

^b^: Hospitalization records were recorded according to the time of admission, and the record of multiple hospitalizations of one patient is not reprocessed.

Abbreviations: PPC, polyene phosphatidyl choline.
