# Supplementary material for: Effectiveness and Economic Evaluation of Polyene Phosphatidyl Choline in Patients With Liver Diseases Based on Real-World Research
Source: Front Pharmacol. 2022 Mar 7;13:806787. doi: 10.3389/fphar.2022.806787 (PMC8940240; doi:10.3389/fphar.2022.806787)
Supplement: Supplementary file 2 [file Table2.DOCX]

Supplementary Table S2. **Baseline information in phase Ⅲ**

| **Categories** | **Medication** | | | | | | |
| --- | --- | --- | --- | --- | --- | --- | --- |
|  | **PPC**  **(N=1595)** | **Glutathione**  **(N=3300)** | **Magnesium Isoglycyrrhizinate**  **(N=3188)** | **PPC+Glutathione**  **(N=1517)** | **PPC+Magnesium Isoglycyrrhizinate**  **(N=1331)** | **PPC+Glutathione+Magnesium Isoglycyrrhizinate**  **(N=1102)** | **Glutathione+Magnesium Isoglycyrrhizinate**  **(4013)** |
| Gender_Male, n (%) | 1123  (70.41%) | 2241(67.91%) | 1911(59.94%) | 1081(71.26%) | 839(63.04%) | 734(66.61%) | 2729(68.0%) |
| Age ^a^, year, median (IQR) | 60 (51~68) | 60 (50~69) | 56 (48~65) | 59 (50~67) | 57 (48~66) | 56 (48~66) | 55 (47~65) |
| First clinical diagnosis ^b^, n (%) |  |  |  |  |  |  |  |
| Postoperation of tumor / liver transplantation | 778  (48.78%) | 1155  (35.0%) | 1296  (40.65%) | 874  (57.61%) | 574  (43.13%) | 554  (50.27%) | 1821  (45.38%) |
| Viral hepatitis | 91  (5.71%) | 285  (8.64%) | 301  (9.44%) | 85  (5.6%) | 69  (5.18%) | 67  (6.08%) | 343  (8.55%) |
| Liver cirrhosis | 60  (3.76%) | 97  (2.94%) | 87  (2.73%) | 64  (4.22%) | 67  (5.03%) | 37  (3.36%) | 119  (2.97%) |
| Space-occupying lesions/postoperative | 114  (7.15%) | 562  (17.03%) | 363  (11.39%) | 91  (6.00%) | 64  (4.81%) | 54  (4.90%) | 374  (9.32%) |
| Abnormal liver function | 456  (28.59%) | 597  (18.09%) | 842  (26.41%) | 335  (22.08%) | 479  (35.99%) | 331  (30.04%) | 1083  (26.99%) |
| Drug-induced liver injury | 7  (0.44%) | 8  (0.24%) | 7  (0.22%) | 3  (0.20%) | 10  (0.75%) | 11  (1.00%) | 28  (0.70%) |
| Autoimmune liver disease | 0(0.00%) | 1(0.03%) | 0(0.00%) | 1(0.07%) | 0(0.00%) | 0(0.00%) | 1(0.02%) |
| Alcoholic liver disease | 1(0.06%) | 2(0.06%) | 3(0.09%) | 0(0.00%) | 0(0.00%) | 0(0.00%) | 0(0.00%) |
| Non alcoholic fatty liver disease | 46  (2.88%) | 486  (14.73%) | 209  (6.56%) | 33  (2.18%) | 39  (2.93%) | 19  (1.72%) | 136  (3.39%) |
| Hepatic encephalopathy | 2(0.13%) | 1(0.03%) | 0(0.00%) | 1(0.07%) | 0(0.00%) | 0(0.00%) | 0(0.00%) |
| Hepatic vascular diseases | 4(0.25%) | 3(0.09%) | 2(0.06%) | 0(0.00%) | 3(0.23%) | 4(0.36%) | 1(0.02%) |
| Non neoplastic diseases of the biliary tract | 25  (1.57%) | 77  (2.33%) | 59  (1.85%) | 27  (1.78%) | 23  (1.73%) | 19  (1.72%) | 86  (2.14%) |
| Others | 11  (0.69%) | 26  (0.79%) | 18  (0.56%) | 3  (0.20%) | 3  (0.23%) | 6  (0.54%) | 21  (0.52%) |
| Liver disease spectrum ^c^, n (%) |  |  |  |  |  |  |  |
| Postoperation of tumor / liver transplantation | 778  (48.78%) | 1155  (35.00%) | 1296  (40.65%) | 874  (57.61%) | 574  (43.13%) | 554  (50.27%) | 1821  (45.38%) |
| Viral hepatitis | 435  (27.27%) | 797  (24.15%) | 927  (29.08%) | 567  (37.38%) | 294  (22.09%) | 318  (28.86%) | 1266  (31.55%) |
| Liver cirrhosis | 323  (20.25%) | 486  (14.73%) | 590  (18.51%) | 463  (30.52%) | 279  (20.96%) | 260  (23.59%) | 896  (22.33%) |
| Space-occupying lesions/postoperative | 222  (13.92%) | 954  (28.91%) | 699  (21.93%) | 267  (17.60%) | 151  (11.34%) | 178  (16.15%) | 995  (24.79%) |
| Abnormal liver function | 478  (29.97%) | 638  (19.33%) | 879  (27.57%) | 376  (24.79%) | 501  (37.64%) | 372  (33.76%) | 1195  (29.78%) |
| Drug-induced liver injury | 7  (0.44%) | 12  (0.36%) | 8  (0.25%) | 7  (0.46%) | 11  (0.83%) | 17  (1.54%) | 33  (0.82%) |
| Autoimmune liver disease | 0(0.0%) | 7(0.21%) | 1(0.03%) | 1(0.07%) | 1(0.08%) | 2(0.18%) | 6(0.15%) |
| Alcoholic liver disease | 3(0.19%) | 6(0.18%) | 5(0.16%) | 3(0.20%) | 0(0.0%) | 4(0.36%) | 8(0.20%) |
| Non alcoholic fatty liver disease | 59  (3.70%) | 560  (16.97%) | 339  (10.63%) | 51  (3.36%) | 69  (5.18%) | 53  (4.81%) | 263  (6.55%) |
| Hepatic encephalopathy | 3(0.19%) | 2(0.06%) | 0(0.0%) | 1(0.07%) | 0(0.0%) | 0(0.0%) | 2(0.05%) |
| Hepatic vascular diseases | 6  (0.38%) | 7  (0.21%) | 17  (0.53%) | 3  (0.20%) | 7  (0.53%) | 8  (0.73%) | 8  (0.20%) |
| Non neoplastic diseases of the biliary tract | 40  (2.51%) | 117  (3.55%) | 106  (3.32%) | 52  (3.43%) | 41  (3.08%) | 37  (3.36%) | 163  (4.06%) |
| Others | 12  (0.75%) | 35  (1.06%) | 44  (1.38%) | 9  (0.59%) | 7  (0.53%) | 8  (0.73%) | 48  (1.20%) |
| Basic chronic disease, n (%) |  |  |  |  |  |  |  |
| Hypertension | 364  (22.82%) | 956  (28.97%) | 574  (18.01%) | 369  (24.32%) | 210  (15.78%) | 207  (18.78%) | 730  (18.19%) |
| Diabetes | 205  (12.85%) | 480  (14.55%) | 337  (10.57%) | 204  (13.45%) | 100  (7.51%) | 120  (10.89%) | 438  (10.91%) |
| Hyperlipidemia | 15  (0.94%) | 30  (0.91%) | 29  (0.91%) | 10  (0.66%) | 5  (0.38%) | 2  (0.18%) | 9  (0.22%) |
| Surgery situation, n (%) |  |  |  |  |  |  |  |
| Non-surgery | 918(57.55%) | 2037(61.73%) | 1525(47.84%) | 752(49.57%) | 849(63.79%) | 624(56.62%) | 1949(48.57%) |
| Surgery may affect liver function (ALT) | 429(26.90%) | 866(26.24%) | 1044(32.75%) | 596(39.29%) | 318(23.89%) | 329(29.85%) | 1350(33.64%) |
| No significant / unknown effect of surgery on liver function (ALT) | 248(15.55%) | 397(12.03%) | 619(19.42%) | 169(11.14%) | 164(12.32%) | 149(13.52%) | 714(17.79%) |

^a^: Age (years) = (admission date in the hospitalization record - date of birth in the patient's information) / 365.25.

^b^: The first clinical diagnosis was based on discharge diagnosis.

^c^: Liver disease spectrum was recorded according to the discharge diagnosis in the hospitalization records (extracting all diagnoses related to the word "hepatic" or "liver").

Abbreviations: PPC, polyene phosphatidyl choline; ALT, alanine transaminase.
