# Supplementary material for: Effectiveness and Economic Evaluation of Polyene Phosphatidyl Choline in Patients With Liver Diseases Based on Real-World Research
Source: Front Pharmacol. 2022 Mar 7;13:806787. doi: 10.3389/fphar.2022.806787 (PMC8940240; doi:10.3389/fphar.2022.806787)
Supplement: Supplementary file 3 [file Table5.DOCX]

**Table S5. Cost minimization analysis in phase Ⅲ (medication costs were reduced by 10%; examination costs were increased by 5%; total efficiency decreased by 10%)**

| **No** | **Medication combination** | **Hospitalization records (N)** | **Total hospitalization costs (Mean, CNY)** | ***p*-value** |
| --- | --- | --- | --- | --- |
| **The whole group** | | | | |
| 1 | PPC | 1528 | 25295.8 | 0.134 |
|  | Magnesium isoglycyrrhizinate | 2299 | 27272.3 |  |
| 2 | PPC+Glutathione | 1509 | 32804.5 | 0.000 |
|  | Glutathione | 2222 | 28084.2 |  |
| 3 | PPC+Magnesium isoglycyrrhizinate | 969 | 28980.6 | 0.000 |
|  | Magnesium isoglycyrrhizinate+Glutathione | 2160 | 34216.5 |  |
| 4 | PPC+Magnesium isoglycyrrhizinate+Glutathione | 1088 | 35723.1 | 0.960 |
|  | Magnesium isoglycyrrhizinate+Glutathione | 2658 | 36058.5 |  |
| **Non-tumor / liver transplantation / postoperative group** | | | | |
| 1 | PPC | 766 | 27285.5 | 0.181 |
|  | Magnesium isoglycyrrhizinate | 1208 | 30319.1 |  |
| 2 | PPC+Glutathione | 622 | 34639.0 | 0.220 |
|  | Magnesium isoglycyrrhizinate+Glutathione | 1181 | 37876.3 |  |
| **Abnormal liver function group** | | | | |
| 1 | PPC | 398 | 32140.5 | 0.712 |
|  | Glutathione | 543 | 37950.7 |  |
| 2 | PPC | 409 | 28864.3 | 0.108 |
|  | Magnesium isoglycyrrhizinate | 409 | 33964.9 |  |
| 3 | PPC+Glutathione | 344 | 39538.0 | 0.000 |
|  | Glutathione | 546 | 33910.5 |  |

Abbreviations: PPC, polyene phosphatidyl choline; CNY, China Yuan.
