# Supplementary material for: Effectiveness and Economic Evaluation of Polyene Phosphatidyl Choline in Patients With Liver Diseases Based on Real-World Research
Source: Front Pharmacol. 2022 Mar 7;13:806787. doi: 10.3389/fphar.2022.806787 (PMC8940240; doi:10.3389/fphar.2022.806787)
Supplement: Supplementary file 4 [file Table3.DOCX]

**Table S3. AST change and test results in different medication combinations**

| **No** | **Medication combination** | **Sample size before PSM, N** | **Sample size after PSM, N** | **AST_recovery** | | **AST_change** | | |
| --- | --- | --- | --- | --- | --- | --- | --- | --- |
|  |  |  |  | **N (%)** | **Chi-square test**  **(p-value)** | **Median** | | **Mann-Whitney U test**  **(p-value)** |
| **The whole group** | | | | | | | | |
| 1 | Glutathione | 3125 | 2653 | 543(47.47%) | 4.9(0.027) | 0 | 1738253.0(0.000) | |
|  | PPC | 1416 | 1412 | 300(42.19%) |  | 0 |  |  |
| 2 | Magnesium isoglycyrrhizinate | 3082 | 2205 | 546(50.09%) | 11.0(0.001) | 0 | 1432717.0(0.023) | |
|  | PPC | 1416 | 1361 | 287(42.02%) |  | 0 |  |  |
| 3 | Magnesium isoglycyrrhizinate | 3082 | 1753 | 514(50.44%) | 0.5(0.502) | -3 | 1166849.0(0.004) | |
|  | PPC+Magnesium isoglycyrrhizinate | 1288 | 1254 | 415(48.88%) |  | -6 |  |  |
| 4 | Glutathione | 3125 | 2051 | 461(46.66%) | 1.6(0.202) | -1 | 1419787.0(0.465) | |
|  | PPC+Glutathione | 1413 | 1405 | 358(43.66%) |  | 0 |  |  |
| 5 | Magnesium isoglycyrrhizinate+Glutathione | 3830 | 2296 | 716(49.93%) | 8.7(0.003) | -7 | 1426978.0(0.000) | |
|  | PPC+Glutathione | 1413 | 1399 | 356(43.47%) |  | 0 |  |  |
| 6 | Glutathione+Magnesium isoglycyrrhizinate | 3830 | 2029 | 611(49.43%) | 1.0(0.314) | -3 | 922014.0(0.343) | |
|  | PPC+Magnesium isoglycyrrhizinate | 1288 | 929 | 283(46.93%) |  | -2 |  |  |
| 7 | Magnesium isoglycyrrhizinate+Glutathione | 3830 | 2502 | 864(52.55%) | 7.2(0.007) | -3 | 1209412.0(0.021) | |
|  | PPC+Magnesium isoglycyrrhizinate+Glutathione | 1031 | 1017 | 327(46.51%) |  | -3 |  |  |
| **Tumor / liver transplantation / postoperative group** | | | | | | | | |
| 1 | Glutathione | 1049 | 660 | 60(22.99%) | 4.1(0.042) | 7 | 207516.0(0.005) | |
|  | PPC | 722 | 690 | 45(16.07%) |  | 12 |  |  |
| 2 | Magnesium isoglycyrrhizinate | 1232 | 633 | 64(26.34%) | 7.5(0.006) | 5 | 173839.5(0.000) | |
|  | PPC | 722 | 631 | 42(16.34%) |  | 12 |  |  |
| 3 | Magnesium isoglycyrrhizinate | 1232 | 715 | 84(26.67%) | 0.01(0.924) | 4 | 186033.5(0.891) | |
|  | PPC+Magnesium isoglycyrrhizinate | 547 | 518 | 70(26.32%) |  | 4 |  |  |
| 4 | Glutathione | 1049 | 761 | 129(33.08%) | 0.2(0.642) | 3 | 289157.5(0.155) | |
|  | PPC+Glutathione | 821 | 793 | 129(31.54%) |  | 4 |  |  |
| 5 | Magnesium isoglycyrrhizinate+Glutathione | 1698 | 1296 | 280(40.00%) | 8.2(0.004) | -2 | 452753.0(0.000) | |
|  | PPC+Glutathione | 821 | 820 | 134(31.53%) |  | 4 |  |  |
| 6 | Glutathione+Magnesium isoglycyrrhizinate | 1698 | 1076 | 159(31.30%) | 8.4(0.004) | 1 | 189261.5(0.000) | |
|  | PPC+Magnesium isoglycyrrhizinate | 547 | 410 | 42(20.49%) |  | 8 |  |  |
| 7 | Magnesium isoglycyrrhizinate+Glutathione | 1698 | 1188 | 228(35.35%) | 0.3(0.558) | -2 | 257361.5(0.003) | |
|  | PPC+Magnesium isoglycyrrhizinate+Glutathione | 507 | 477 | 91(33.33%) |  | 1 |  |  |
| **Non-tumor / liver transplantation / postoperative group** | | | | | | | | |
| 1 | Glutathione | 2076 | 1200 | 367(57.17%) | 0.3(0.596) | -4 | 443025.5(0.011) | |
|  | PPC | 694 | 690 | 247(58.81%) |  | -8 |  |  |
| 2 | Magnesium isoglycyrrhizinate | 1850 | 1177 | 456(63.42%) | 2.5(0.116) | -6 | 400125.0(0.215) | |
|  | PPC | 694 | 657 | 234(58.65%) |  | -7 |  |  |
| 3 | Magnesium isoglycyrrhizinate | 1850 | 969 | 431(62.28%) | 2.0(0.161) | -15 | 376865.5(0.000) | |
|  | PPC+Magnesium isoglycyrrhizinate | 741 | 704 | 324(58.38%) |  | -24 |  |  |
| 4 | Glutathione | 2076 | 1225 | 371(57.16%) | 0.01(0.910) | -3 | 372192.5(0.010) | |
|  | PPC+Glutathione | 592 | 565 | 213(56.80%) |  | -7 |  |  |
| 5 | Magnesium isoglycyrrhizinate+Glutathione | 2132 | 1123 | 486(60.30%) | 2.1(0.151) | -10 | 325359.0(0.618) | |
|  | PPC+Glutathione | 592 | 571 | 217(55.93%) |  | -8 |  |  |
| 6 | Glutathione+Magnesium isoglycyrrhizinate | 2132 | 927 | 431(60.88%) | 0.7(0.415) | -8 | 254506.0(0.005) | |
|  | PPC+Magnesium isoglycyrrhizinate | 741 | 504 | 227(58.35%) |  | -18 |  |  |
| 7 | Magnesium isoglycyrrhizinate+Glutathione | 2132 | 1236 | 575(60.46%) | 1.9(0.166) | -2 | 324047.5(0.507) | |
|  | PPC+Magnesium isoglycyrrhizinate+Glutathione | 524 | 514 | 232(56.45%) |  | -7 |  |  |
| **Abnormal liver function group** | | | | | | | | |
| 1 | Glutathione | 603 | 518 | 225(54.88%) | 0.7(0.393) | -14 | 92202.5(0.030) | |
|  | PPC | 372 | 327 | 160(58.18%) |  | -19 |  |  |
| 2 | Magnesium isoglycyrrhizinate | 860 | 401 | 202(60.66%) | 0.4(0.535) | -24 | 65096.0(0.848) | |
|  | PPC | 372 | 322 | 160(58.18%) |  | -20 |  |  |
| 3 | Magnesium isoglycyrrhizinate | 860 | 467 | 261(63.66%) | 1.7(0.187) | -28 | 114825.0(0.039) | |
|  | PPC+Magnesium isoglycyrrhizinate | 487 | 456 | 245(59.18%) |  | -34 |  |  |
| 4 | Glutathione | 603 | 517 | 218(53.83%) | 0.2(0.645) | -12 | 87151.5(0.043) | |
|  | PPC+Glutathione | 341 | 311 | 148(55.64%) |  | -23 |  |  |
| 5 | Magnesium isoglycyrrhizinate+Glutathione | 1169 | 705 | 364(58.62%) | 1.3(0.251) | -13 | 126534.0(0.007) | |
|  | PPC+Glutathione | 341 | 325 | 151(54.51%) |  | -23 |  |  |
| 6 | Glutathione+Magnesium isoglycyrrhizinate | 1169 | 506 | 271(60.63%) | 0.0(0.993) | -16 | 95265.0(0.004) | |
|  | PPC+Magnesium isoglycyrrhizinate | 487 | 337 | 183(60.60%) |  | -27 |  |  |
| 7 | Magnesium isoglycyrrhizinate+Glutathione | 1169 | 787 | 427(61.17%) | 9.3(0.002) | 6 | 145972.5(0.090) | |
|  | PPC+Magnesium isoglycyrrhizinate+Glutathione | 360 | 349 | 160(50.96%) |  | -5 |  |  |
| **Viral hepatitis group** | | | | | | | | |
| 1 | Glutathione | 752 | 594 | 72(28.46%) | 1.9(0.173) | 4 | 109384.5(0.001) | |
|  | PPC | 420 | 419 | 40(22.60%) |  | 9 |  |  |
| 2 | Magnesium isoglycyrrhizinate | 900 | 554 | 49(24.87%) | 0.4(0.527) | 8 | 98697.0(0.155) | |
|  | PPC | 420 | 377 | 35(22.01%) |  | 10 |  |  |
| 3 | Magnesium isoglycyrrhizinate | 900 | 407 | 41(25.62%) | 0.002(0.961) | 4 | 57394.0(0.415) | |
|  | PPC+Magnesium isoglycyrrhizinate | 284 | 272 | 34(25.37%) |  | 5 |  |  |
| 4 | Glutathione | 752 | 451 | 66(29.46%) | 0.4(0.515) | 4 | 103082.0(0.657) | |
|  | PPC+Glutathione | 542 | 465 | 79(32.24%) |  | 5 |  |  |
| 5 | Magnesium isoglycyrrhizinate+Glutathione | 1205 | 811 | 173(39.68%) | 2.7(0.103) | -1 | 199652.0(0.005) | |
|  | PPC+Glutathione | 542 | 541 | 102(33.77%) |  | 4 |  |  |
| 6 | Glutathione+Magnesium isoglycyrrhizinate | 1205 | 690 | 124(35.73%) | 7.4(0.006) | 1 | 76725.0(0.010) | |
|  | PPC+Magnesium isoglycyrrhizinate | 284 | 250 | 30(22.73%) |  | 7 |  |  |
| 7 | Magnesium isoglycyrrhizinate+Glutathione | 1205 | 764 | 169(39.12%) | 4.0(0.045) | 0 | 105261.5(0.156) | |
|  | PPC+Magnesium isoglycyrrhizinate+Glutathione | 293 | 292 | 52(30.41%) |  | 2 |  |  |
| **Liver cirrhosis group** | | | | | | | | |
| 1 | Glutathione | 455 | 293 | 28(23.33%) | 0.1(0.728) | 4 | 41374.5(0.492) | |
|  | PPC | 300 | 292 | 24(21.43%) |  | 4 |  |  |
| 2 | Magnesium isoglycyrrhizinate | 574 | 253 | 43(35.83%) | 5.8(0.016) | 4 | 29154.5(0.149) | |
|  | PPC | 300 | 249 | 20(20.83%) |  | 6 |  |  |
| 3 | Magnesium isoglycyrrhizinate | 574 | 320 | 44(29.93%) | 1.9(0.173) | 4 | 40896.5(0.219) | |
|  | PPC+Magnesium isoglycyrrhizinate | 267 | 241 | 29(22.66%) |  | 4 |  |  |
| 4 | Glutathione | 455 | 342 | 44(26.19%) | 0.4(0.541) | 3 | 59417.0(0.718) | |
|  | PPC+Glutathione | 433 | 342 | 51(29.14%) |  | 3 |  |  |
| 5 | Magnesium isoglycyrrhizinate+Glutathione | 853 | 640 | 130(37.46%) | 4.1(0.042) | 0 | 127840.0(0.031) | |
|  | PPC+Glutathione | 433 | 433 | 66(29.20%) |  | 3 |  |  |
| 6 | Glutathione+Magnesium isoglycyrrhizinate | 853 | 549 | 96(34.66%) | 9.1(0.003) | 2 | 53501.5(0.125) | |
|  | PPC+Magnesium isoglycyrrhizinate | 267 | 210 | 20(18.87%) |  | 6 |  |  |
| 7 | Magnesium isoglycyrrhizinate+Glutathione | 853 | 569 | 107(36.15%) | 1.8(0.176) | 1 | 65729.0(0.352) | |
|  | PPC+Magnesium isoglycyrrhizinate+Glutathione | 241 | 241 | 36(29.27%) |  | 2 |  |  |

Notes: the data size of some medication combinations in the non-tumor-abnormal liver function group was too small to be included in the analysis.

Abbreviations: AST, aspartate aminotransferase; PSM, propensity score matching; PPC, polyene phosphatidyl choline. AST_change indicates AST level relative to baseline after treatment; AST_recovery indicates cases with abnormal AST that changes to normal range (<40 U/L) after treatment.
