# Supplementary material for: Effectiveness and Economic Evaluation of Polyene Phosphatidyl Choline in Patients With Liver Diseases Based on Real-World Research
Source: Front Pharmacol. 2022 Mar 7;13:806787. doi: 10.3389/fphar.2022.806787 (PMC8940240; doi:10.3389/fphar.2022.806787)
Supplement: Supplementary file 5 [file Table4.DOCX]

**Table S4. TBil change value and test results in different medication combinations**

| **No** | **Medication combination** | **Sample size before PSM, N** | **Sample size after PSM, N** | **TBil_recovery** | | **TBil_change** | |
| --- | --- | --- | --- | --- | --- | --- | --- |
|  |  |  |  | **N (%)** | **Chi-square test**  **(p-value)** | **Median** | **Mann-Whitney U test**  **(p-value)** |
| **The whole group** | | | | | | | |
| 1 | Glutathione | 3288 | 2812 | 338(36.62%) | 5.6(0.018) | -1 | 1839283.5(0.000) |
|  | PPC | 1527 | 1523 | 184(30.72%) |  | 1 |  |
| 2 | Magnesium isoglycyrrhizinate | 3180 | 2296 | 253(35.14%) | 2.7(0.103) | 1 | 1637641.0(0.197) |
|  | PPC | 1527 | 1463 | 177(30.84%) |  | 1 |  |
| 3 | Magnesium isoglycyrrhizinate | 3180 | 1820 | 217(34.89%) | 0.5(0.462) | 0 | 1212323.5(0.138) |
|  | PPC+Magnesium isoglycyrrhizinate | 1327 | 1292 | 183(32.85%) |  | 0 |  |
| 4 | Glutathione | 3288 | 2212 | 296(35.20%) | 3.9(0.049) | -1 | 1474790.5(0.000) |
|  | PPC+Glutathione | 1505 | 1497 | 198(30.37%) |  | 1 |  |
| 5 | Magnesium isoglycyrrhizinate+Glutathione | 4011 | 2471 | 390(33.22%) | 1.7(0.196) | -1 | 1679571.5(0.000) |
|  | PPC+Glutathione | 1505 | 1491 | 198(30.28%) |  | 1 |  |
| 6 | Glutathione+Magnesium isoglycyrrhizinate | 4011 | 2158 | 281(31.47%) | 0.1(0.790) | 0 | 994072.0(0.034) |
|  | PPC+Magnesium isoglycyrrhizinate | 1327 | 967 | 126(30.73%) |  | 1 |  |
| 7 | Magnesium isoglycyrrhizinate+Glutathione | 4011 | 2656 | 380(33.75%) | 1.6(0.207) | 0 | 1335009.0(0.000) |
|  | PPC+Magnesium isoglycyrrhizinate+Glutathione | 1100 | 1086 | 131(30.39%) |  | 0 |  |
| **Tumor / liver transplantation / postoperative group** | | | | | | | |
| 1 | Glutathione | 1155 | 746 | 54(19.85%) | 1.8(0.184) | 2 | 221796.0(0.000) |
|  | PPC | 778 | 746 | 47(15.61%) |  | 4 |  |
| 2 | Magnesium isoglycyrrhizinate | 1295 | 677 | 51(23.18%) | 2.3(0.130) | 2 | 192794.5(0.000) |
|  | PPC | 778 | 678 | 46(17.62%) |  | 4 |  |
| 3 | Magnesium isoglycyrrhizinate | 1295 | 760 | 68(26.77%) | 0.9(0.349) | 2 | 193535.5(0.056) |
|  | PPC+Magnesium isoglycyrrhizinate | 574 | 543 | 49(23.00%) |  | 3 |  |
| 4 | Glutathione | 1155 | 844 | 98(26.49%) | 2.6(0.104) | 1 | 295096.0(0.000) |
|  | PPC+Glutathione | 874 | 844 | 78(21.37%) |  | 3 |  |
| 5 | Magnesium isoglycyrrhizinate+Glutathione | 1821 | 1415 | 193(27.30%) | 4.4(0.037) | 1 | 533583.0(0.000) |
|  | PPC+Glutathione | 874 | 873 | 82(21.52%) |  | 3 |  |
| 6 | Glutathione+Magnesium isoglycyrrhizinate | 1821 | 1189 | 111(21.02%) | 2.6(0.108) | 2 | 223777.5(0.000) |
|  | PPC+Magnesium isoglycyrrhizinate | 574 | 437 | 26(15.38%) |  | 5 |  |
| 7 | Magnesium isoglycyrrhizinate+Glutathione | 1821 | 1300 | 146(23.78%) | 0.02(0.890) | 1 | 294505.0(0.000) |
|  | PPC+Magnesium isoglycyrrhizinate+Glutathione | 554 | 523 | 52(23.32%) |  | 3 |  |
| **Non-tumor / liver transplantation / postoperative group** | | | | | | | |
| 1 | Glutathione | 2133 | 1249 | 175(45.93%) | 0.04(0.845) | -2 | 469351.5(0.742) |
|  | PPC | 749 | 745 | 131(45.17%) |  | -2 |  |
| 2 | Magnesium isoglycyrrhizinate | 1885 | 1204 | 170(43.04%) | 0.1(0.759) | 0 | 467491.0(0.000) |
|  | PPC | 749 | 707 | 119(44.24%) |  | -2 |  |
| 3 | Magnesium isoglycyrrhizinate | 1885 | 994 | 153(41.69%) | 0.4(0.538) | -1 | 386641.5(0.002) |
|  | PPC+Magnesium isoglycyrrhizinate | 753 | 716 | 128(39.38%) |  | -2 |  |
| 4 | Glutathione | 2133 | 1280 | 182(44.83%) | 0.2(0.621) | -2 | 389278.5(0.673) |
|  | PPC+Glutathione | 631 | 601 | 108(42.86%) |  | -2 |  |
| 5 | Magnesium isoglycyrrhizinate+Glutathione | 2190 | 1180 | 211(43.78%) | 0.1(0.732) | -2 | 363410.0(0.735) |
|  | PPC+Glutathione | 631 | 610 | 113(42.48%) |  | -2 |  |
| 6 | Glutathione+Magnesium isoglycyrrhizinate | 2190 | 968 | 152(43.68%) | 0.2(0.647) | -1 | 255201.0(0.449) |
|  | PPC+Magnesium isoglycyrrhizinate | 753 | 515 | 99(41.77%) |  | -2 |  |
| 7 | Magnesium isoglycyrrhizinate+Glutathione | 2190 | 1283 | 220(44.53%) | 2.6(0.110) | -1 | 339252.0(0.653) |
|  | PPC+Magnesium isoglycyrrhizinate+Glutathione | 546 | 536 | 73(37.82%) |  | -1 |  |
| **Abnormal liver function group** | | | | | | | |
| 1 | Glutathione | 630 | 537 | 105(50.00%) | 0.4(0.533) | -2 | 100599.5(0.210) |
|  | PPC | 412 | 357 | 70(46.67%) |  | -3 |  |
| 2 | Magnesium isoglycyrrhizinate | 872 | 407 | 60(37.04%) | 3.7(0.053) | -1 | 79915.5(0.017) |
|  | PPC | 412 | 357 | 70(47.95%) |  | -3 |  |
| 3 | Magnesium isoglycyrrhizinate | 872 | 467 | 82(45.05%) | 2.3(0.131) | -1 | 116604.5(0.058) |
|  | PPC+Magnesium isoglycyrrhizinate | 497 | 466 | 80(37.56%) |  | -2 |  |
| 4 | Glutathione | 630 | 539 | 103(47.25%) | 0.7(0.400) | -2 | 88109.0(0.651) |
|  | PPC+Glutathione | 364 | 333 | 62(42.76%) |  | -2 |  |
| 5 | Magnesium isoglycyrrhizinate+Glutathione | 1193 | 728 | 127(45.20%) | 0.3(0.587) | -1 | 131855.5(0.277) |
|  | PPC+Glutathione | 364 | 348 | 65(42.48%) |  | -2 |  |
| 6 | Glutathione+Magnesium isoglycyrrhizinate | 1193 | 510 | 78(49.68%) | 6.1(0.014) | -1 | 95684.5(0.036) |
|  | PPC+Magnesium isoglycyrrhizinate | 497 | 346 | 56(35.90%) |  | -2 |  |
| 7 | Magnesium isoglycyrrhizinate+Glutathione | 1193 | 799 | 123(47.86%) | 3.9(0.048) | 0 | 141814.0(0.760) |
|  | PPC+Magnesium isoglycyrrhizinate+Glutathione | 370 | 359 | 46(37.10%) |  | -1 |  |
| **Viral hepatitis group** | | | | | | | |
| 1 | Glutathione | 796 | 637 | 66(27.27%) | 1.5(0.222) | 1 | 105842.5(0.000) |
|  | PPC | 435 | 434 | 39(22.03%) |  | 4 |  |
| 2 | Magnesium isoglycyrrhizinate | 927 | 577 | 35(20.11%) | 0.3(0.564) | 3 | 103908.5(0.043) |
|  | PPC | 435 | 390 | 35(22.73%) |  | 4 |  |
| 3 | Magnesium isoglycyrrhizinate | 927 | 421 | 34(25.00%) | 4.5(0.034) | 3 | 54551.0(0.068) |
|  | PPC+Magnesium isoglycyrrhizinate | 294 | 282 | 15(14.02%) |  | 5 |  |
| 4 | Glutathione | 796 | 486 | 61(29.90%) | 0.4(0.507) | 0 | 96307.5(0.000) |
|  | PPC+Glutathione | 567 | 487 | 58(26.98%) |  | 4 |  |
| 5 | Magnesium isoglycyrrhizinate+Glutathione | 1266 | 872 | 118(28.03%) | 0.04(0.838) | 2 | 223646.0(0.003) |
|  | PPC+Glutathione | 567 | 566 | 71(27.31%) |  | 3 |  |
| 6 | Glutathione+Magnesium isoglycyrrhizinate | 1266 | 735 | 90(25.94%) | 8.2(0.004) | 2 | 83663.0(0.003) |
|  | PPC+Magnesium isoglycyrrhizinate | 294 | 260 | 13(12.50%) |  | 5 |  |
| 7 | Magnesium isoglycyrrhizinate+Glutathione | 1266 | 823 | 98(25.65%) | 1.1(0.301) | 1 | 119642.5(0.037) |
|  | PPC+Magnesium isoglycyrrhizinate+Glutathione | 318 | 316 | 30(21.28%) |  | 2 |  |
| **Liver cirrhosis group** | | | | | | | |
| 1 | Glutathione | 485 | 313 | 28(18.18%) | 0.02(0.895) | 1 | 40944.5(0.000) |
|  | PPC | 323 | 314 | 28(17.61%) |  | 3 |  |
| 2 | Magnesium isoglycyrrhizinate | 590 | 265 | 21(17.07%) | 0.2(0.639) | 3 | 32471.0(0.134) |
|  | PPC | 323 | 265 | 20(14.93%) |  | 4 |  |
| 3 | Magnesium isoglycyrrhizinate | 590 | 333 | 27(18.12%) | 0.3(0.558) | 3 | 39566.5(0.238) |
|  | PPC+Magnesium isoglycyrrhizinate | 279 | 252 | 19(15.45%) |  | 5 |  |
| 4 | Glutathione | 485 | 365 | 35(17.95%) | 0.5(0.488) | 0 | 55497.5(0.000) |
|  | PPC+Glutathione | 463 | 366 | 36(20.81%) |  | 3 |  |
| 5 | Magnesium isoglycyrrhizinate+Glutathione | 896 | 683 | 89(23.54%) | 1.4(0.231) | 2 | 142166.0(0.004) |
|  | PPC+Glutathione | 463 | 462 | 44(19.38%) |  | 4 |  |
| 6 | Glutathione+Magnesium isoglycyrrhizinate | 896 | 589 | 66(21.02%) | 1.6(0.210) | 3 | 57687.0(0.013) |
|  | PPC+Magnesium isoglycyrrhizinate | 279 | 221 | 16(15.38%) |  | 5 |  |
| 7 | Magnesium isoglycyrrhizinate+Glutathione | 896 | 608 | 64(19.39%) | 0.5(0.482) | 2 | 73632.0(0.110) |
|  | PPC+Magnesium isoglycyrrhizinate+Glutathione | 260 | 260 | 21(16.54%) |  | 3 |  |

Notes: the data size of some medication combinations in the non-tumor-abnormal liver function group was too small to be included in the analysis.

Abbreviations: TBil, total bilirubin; PSM, propensity score matching; PPC, polyene phosphatidyl choline. TBil_change indicates TBil level relative to baseline after treatment; TBil_recovery indicates cases with abnormal TBil that changes to normal range(<17.1μmol/L) after treatment.
