# Supplementary material for: Effectiveness and Economic Evaluation of Polyene Phosphatidyl Choline in Patients With Liver Diseases Based on Real-World Research
Source: Front Pharmacol. 2022 Mar 7;13:806787. doi: 10.3389/fphar.2022.806787 (PMC8940240; doi:10.3389/fphar.2022.806787)
Supplement: Supplementary file 6 [file Table6.DOCX]

**Table S6. Cost-effectiveness analysis in phase Ⅲ (medication costs were reduced by 10%; examination costs were increased by 5%; total efficiency decreased by 10%)**

| **No** | **Medication combination** | **Hospitalization records (N)** | **Effective records (N)** | **Costs (CNY)** | **Total effective rate (effectiveness, E%)** | **Cost effectiveness ratio (C/E)** | **Incremental cost effectiveness ratio (△C/△E)** |
| --- | --- | --- | --- | --- | --- | --- | --- |
| **The whole group** | | | | | | | |
| 1 | PPC | 1591 | 424 | 28786.1 | 26.65% | 108015.8 | 32400.0 |
|  | Glutathione | 2823 | 700 | 28186.7 | 24.8% | 113672.9 |  |
| 2 | PPC+Magnesium isoglycyrrhizinate | 1296 | 459 | 24935.5 | 35.42% | 70406.1 | -6633.9 |
|  | Magnesium isoglycyrrhizinate | 1828 | 586 | 25158.4 | 32.06% | 78480.5 |  |
| 3 | PPC+Glutathione | 1503 | 527 | 31573.3 | 35.06% | 90046.8 | -141201.9 |
|  | Magnesium isoglycyrrhizinate+Glutathione | 2473 | 850 | 32547.6 | 34.37% | 94694.4 |  |
| **Non-tumor / liver transplantation / postoperative group** | | | | | | | |
| 1 | PPC | 813 | 354 | 30489.8 | 43.54% | 70023.2 | -21560.6 |
|  | Glutathione | 1260 | 454 | 32109 | 36.03% | 89113.1 |  |
| 2 | PPC + Magnesium isoglycyrrhizinate | 720 | 350 | 26701 | 48.61% | 54927.8 | -41735.6 |
|  | Magnesium isoglycyrrhizinate | 1000 | 460 | 27790.3 | 46.0% | 60413.7 |  |
| 3 | PPC+Glutathione | 613 | 246 | 35075.1 | 40.13% | 87402.6 | 60756.0 |
|  | Glutathione | 1291 | 454 | 32061.6 | 35.17% | 91170.8 |  |
| 4 | PPC + Magnesium isoglycyrrhizinate | 517 | 248 | 31214.8 | 47.97% | 65072.8 | -191525.4 |
|  | Magnesium isoglycyrrhizinate+Glutathione | 969 | 442 | 35734.8 | 45.61% | 78341.7 |  |
| 5 | PPC+Magnesium isoglycyrrhizinate+Glutathione | 538 | 258 | 42763.9 | 47.96% | 89174.3 | 100064.8 |
|  | Magnesium isoglycyrrhizinate+Glutathione | 1284 | 602 | 41683.2 | 46.88% | 88905.7 |  |
| **Abnormal liver function group** | | | | | | | |
| 1 | PPC+Magnesium isoglycyrrhizinate | 470 | 255 | 25683.8 | 54.26% | 47338.8 | 163707.5 |
|  | Magnesium isoglycyrrhizinate | 470 | 265 | 29154.4 | 56.38% | 51707.8 |  |
| 2 | PPC+Glutathione | 360 | 180 | 38715.7 | 50.0% | 77431.4 | 170279.0 |
|  | Magnesium isoglycyrrhizinate+Glutathione | 730 | 382 | 42683.2 | 52.33% | 81567.4 |  |
| 3 | PPC+Magnesium isoglycyrrhizinate | 348 | 187 | 30475 | 53.74% | 56712.8 | -401969.2 |
|  | Magnesium isoglycyrrhizinate+Glutathione | 511 | 263 | 39599.7 | 51.47% | 76940.9 |  |
| 4 | PPC+Magnesium isoglycyrrhizinate+Glutathione | 361 | 169 | 39418.3 | 46.81% | 84201.2 | 156360.8 |
|  | Magnesium isoglycyrrhizinate+Glutathione | 800 | 390 | 42451.7 | 48.75% | 87080.4 |  |

Abbreviations: PPC, polyene phosphatidyl choline; CNY, China Yuan; C, cost; E, effectiveness.
